# Trade-Offs in Growth Performance, Serum Biochemistry, and Fillet Amino Acid Profiles of Red Tilapia Fed Graded Levels of *Azolla microphylla* Meal in a Fishmeal Replacement Blend, with Exploratory Observations on Intestinal Microbiota

**DOI:** 10.3390/vetsci13101039

**Published:** 2026-10-02

**Authors:** Panitnart Auputinan, Nantapong Sumket, Somchart Tana, Payungsuk Intawicha, Phanit Srisuttha, Phadungnaree Saenmuang, Sontaya Sookying, Dutrudi Panprommin, Paiboon Panase

**Affiliations:** 1 Division of Biotechnology, School of Agriculture and Natural Resources, University of Phayao, Phayao 56000, Thailand; panitnart.au@up.ac.th (P.A.); packpack2256@gmail.com (N.S.); 2 Division of Animal Science, School of Agriculture and Natural Resources, University of Phayao, Phayao 56000, Thailand; somchart.ta@up.ac.th (S.T.); payungsuk.in@up.ac.th (P.I.); 3 Department of Applied Thai Traditional Medicine, School of Public Health, University of Phayao, Phayao 56000, Thailand; phanit.sr@up.ac.th (P.S.); phadungnaree.se@up.ac.th (P.S.); 4 Division of Pharmacy and Technology, Department of Pharmaceutical Care, School of Pharmaceutical Sciences, University of Phayao, Phayao 56000, Thailand; sontaya.so@up.ac.th; 5 Division of Fisheries, School of Agriculture and Natural Resources, University of Phayao, Phayao 56000, Thailand; dutrudeep@yahoo.com; 6 Unit of Excellence “Physiology for the Sustainable Production and Management of Aquatic Animals”, Division of Fisheries, School of Agriculture and Natural Resources, University of Phayao, Phayao 56000, Thailand

**Keywords:** *Azolla microphylla*, fishmeal replacement, red tilapia, fillet amino acids, intestinal microbiota, *Aeromonas hydrophila*

## Abstract

This study evaluated the replacement of commercial fishmeal with a combination of *Azolla microphylla* meal (AMM) and soybean meal in red tilapia (*Oreochromis* spp.) diets at 0%, 25%, 50%, and 75% fishmeal replacement over 60 days, followed by a sublethal *Aeromonas hydrophila* exposure. Soybean meal was increased alongside AMM to maintain isonitrogenous and isoenergetic diets; therefore, the observed responses reflect the combined replacement of fishmeal rather than the effect of AMM alone. Growth performance (WG, ADG, SGR) progressively declined and FCR increased with higher fishmeal replacement levels (*p* < 0.05). Serum aspartate transaminase (AST) rose and alkaline phosphatase (ALP) fell at elevated replacement levels. Fillet total essential amino acids were significantly reduced only at 75% fishmeal replacement, indicating that moderate substitution (up to 50%) preserves nutritional quality. As an exploratory, descriptive endpoint, the intestinal microbiota was profiled using pooled samples (*n* = 1 composite per treatment per time point), precluding statistical inference. Within the pooled post-challenge profiles, *Cetobacterium* represented a high relative proportion in T1–T3, whereas the pooled T4 profile showed a more heterogeneous taxonomic composition. Because no independent microbiota replicates were available, these observations are descriptive and hypothesis-generating rather than evidence of dietary or challenge effects. Overall, fishmeal replacement above 25% impaired growth, whereas fillet total essential amino acids were maintained through 50% replacement. These effects should be interpreted as the combined outcome of replacing fishmeal with AMM and soybean meal. The pooled microbiota profiles provide preliminary observations for future replicated studies.

## Simple Summary

Red tilapia is one of the most important fish for human food worldwide, but its farming relies heavily on fishmeal, which is expensive and becoming scarce. This study tested whether a water fern called *Azolla microphylla* could replace fishmeal in red tilapia diets. Fish were fed with four different diets, where a combination of *Azolla microphylla* meal and soybean meal replaced 0%, 25%, 50%, or 75% of the fishmeal for 60 days. The results showed that fish grew more slowly and needed more feed to gain weight when fishmeal replacement exceeded 25%. However, the nutritional quality of the fish meats, specifically its essential amino acids, remained good even when the combination replaced up to 50% of the fishmeal. Only at the highest replacement level (75%) did the meat quality decline. The study also explored the gut bacteria of the fish but found that more research is needed to draw firm conclusions. Overall, this research suggests that a combination of *Azolla microphylla* meal and soybean meal can replace up to half of the fishmeal in red tilapia feed without reducing the nutritional value of the fish, offering a more sustainable and cost-effective option for fish farmers.

## 1. Introduction

The red tilapia (*Oreochromis* spp.) is a globally significant aquaculture species, accounting for approximately 80% of total tilapia production [1]. Valued for its rapid growth, environmental resilience, and disease resistance, this hybrid also benefits from a market-preferred reddish-orange pigmentation that enhances its consumer appeal [2,3,4]. As the world’s second most farmed fish, tilapia is a crucial pillar of food security and economic development, especially in low- and middle-income countries [5,6]. However, the sustainable expansion of this vital industry is constrained by its reliance on fishmeal, a costly and finite marine resource. Escalating prices driven by limited supply and high demand threaten both the profitability and environmental sustainability of tilapia farming [7,8]. Consequently, identifying sustainable, cost-effective alternative protein sources has become a major research imperative [9,10]. The aquatic fern *Azolla microphylla* has emerged as one such promising candidate. Characterized by high biomass productivity and a favorable nutritional profile containing 13–30% crude protein, essential amino acids, vitamins, and minerals, it presents a compelling plant-based alternative [11,12]. Studies in various fish species, including tilapia, report that Azolla can be incorporated at low to moderate dietary levels without compromising growth [13]. Conversely, other studies report that inclusion levels exceeding 5% adversely affect growth performance, feed efficiency, and health, largely due to anti-nutritional factors and reduced nutrient digestibility [7]. These contradictory findings underscore persistent constraints on its widespread use as a fishmeal substitute. Importantly, existing research has predominantly focused on basic growth and feed utilization metrics, leaving significant gaps in understanding the underlying physiological and nutritional consequences of Azolla inclusion. A comprehensive evaluation is lacking, particularly regarding its impact on (1) key serum biochemical indices reflecting metabolic health, and (2) the final product quality, specifically the fillet’s fatty acid and amino acid profiles. These nutritional components are vital not only for fish health but are also key determinants of the fillet’s value for human consumers, directly affecting its marketability and health benefits [14,15].

In this study, fishmeal was replaced by a combination of AMM and soybean meal, a common plant protein source in tilapia diets. Soybean meal was increased alongside AMM to maintain isonitrogenous and isoenergetic diets, reflecting practical feed formulation. However, this strategy introduces a confounding factor, as the observed effects cannot be attributed exclusively to AMM. Replacement levels of 0%, 25%, 50%, and 75% were selected based on previous studies on Azolla in tilapia and other fish species, which report beneficial or neutral effects at low inclusion levels and adverse effects above approximately 25–50% [7,10].

The primary objective of this study was to evaluate growth performance, serum biochemical indices, and fillet amino acid profiles of red tilapia fed the experimental fishmeal-replacement diets. Alongside these host-level outcomes, a secondary exploratory component asked which bacterial taxa and community characteristics were present in pooled intestinal samples from fish receiving the same diets, before and after sublethal *A. hydrophila* exposure. Taxonomic composition, diversity, ASV overlap, and predicted functional profiles were examined to document different attributes of these pooled communities and formulate questions for future replicated studies. Each treatment-by-time point was represented by one pooled sample (*n* = 1); therefore, this component provides descriptive observations and cannot establish dietary or exposure effects, or microbial explanations for the primary outcomes.

## 2. Materials and Methods

### 2.1. Ethical Statement

The scientific procedures were conducted according to guidelines for the animal care and use of the University of Phayao and approved by the Committee of Institutional Animal Care, University of Phayao, Thailand (ID: 1-025-68; approval date: 8 October 2025).

### 2.2. Fish Feed Formulations and Experimental Design

Four isonitrogenous diets were formulated by substituting commercial fish meal (CFM) with a combination of *Azolla microphylla* meal (AMM) and soybean meal at 0% (control), 25%, 50%, and 75% of fishmeal. As fishmeal decreased, both AMM and soybean meal increased proportionally to maintain dietary protein and energy levels (Table 1). Soybean meal was included as a co-replacement to maintain isonitrogenous conditions. Therefore, fishmeal was replaced by a combination of AMM and soybean meal rather than by AMM alone, and the observed effects reflect this combined replacement strategy. The proximate composition of the four experimental diets is presented in Table 1. During the experimental phase, red tilapia averaging 13.52 ± 0.17 g were purchased from a commercial fish farm in Chiang Rai Province, Thailand. The fish had a two-week acclimation period in a net cage (3m × 5m × 0.8 m) situated in an earthen pond. Water quality parameters were systematically assessed at 7:00 a.m. throughout the acclimation phase, with temperature stabilized at 30.5 ± 1.51 °C, dissolved oxygen (DO) content at 7.4 ± 0.32 mg/L, and pH at 7.2 ± 0.21, as determined using multi-probes (HORIBA, U50 series, Kyoto, Japan). The twelve cages were randomly assigned to positions within the same earthen pond using a completely randomized design (CRD), with three replicate cages per dietary treatment. Housing all cages in a single pond ensured that all treatments were exposed to the same water source and ambient environmental conditions, thereby minimizing between-pond variation. A total of 360 healthy fish were allocated into four triplicate groups, each including 30 fish per replication. The groups were raised in twelve net cages (1 × 2 × 0.5 m), all situated within the same earthen pond. Each group was administered their designated diet, as previously outlined, twice daily at 5% of their body weight per day, throughout a duration of 60 days.

### 2.3. Biochemical Composition Analysis of Azolla microphylla Meal (AMM), Commercial Fish Meal (CFM) and the Four Experimental Diets

The *A. microphylla* obtained from the local agricultural farm in Phayao province, alongside the commercial fish meal sourced from an agricultural supply store in Chiang Mai, Thailand. Proximate analysis was performed, including evaluations of moisture, ash, crude protein, and crude fat, utilizing the standardized procedures specified in AOAC (2006) [16]. The Kjeldahl distillation method was utilized to discover crude protein content, whereas crude lipid content was evaluated by Soxhlet extraction, and ash content was measured utilizing drying techniques. The results from these studies are displayed in Table 2. The amino acid and fatty acid profiles of the AMM, CFM and the four experimental diets were analyzed, and amino acid profiles were evaluated using a Bio 30+ series amino acid analyzer from Biochrom Ltd., Cambridge, UK, in accordance with the in-house technique TE-CH-372, which is based on Official Journal of the European Communities (1998) [17]. Limits of detection (LOD) were: cystine, and methionine 100.00 mg/100 g; hydroxyproline 200.00 mg/100 g. The analysis of fatty acid profiles was conducted using Gas Chromatography (GC) according to the in-house technique TE-CH-208, based on AOAC (2019) [18] 996.06 standards, with an LOD of 0.01 g/100 g for individual fatty acids. The Analytical Service of the Central Laboratory (Chiangmai, Thailand) Co., Ltd., conducted both analyses in the Chiangmai branch.

### 2.4. Growth Performance and Survival

Red tilapia in each cage was weighed at 15-day intervals to adjust feed levels and evaluate growth performance. The growth metrics encompassed weight gain (WG), average daily gain (ADG), specified growth rate (SGR), feed conversion ratio (FCR), and survival rate (SR). The computations for these parameters were executed utilizing the supplied formulae [19]:

WG (g) = final weight (g) − initial weight (g)

ADG (g/day) = [{final weight (g) − initial weight (g)}]/experimental days

SGR (%/day) = [{ln final weight (g) − ln initial weight (g)}/experimental days] × 100

FCR = total feed fed (g)/weight gain (g)

SR (%) = [number of survived fish/initial number of fish] × 100

### 2.5. Serum Biochemistry Study

On the 60th day of the feeding study, serum biochemistry was analyzed. Following the evaluation of growth indices and survival rates, three fish were randomly selected from each replicate cage (nine fish per treatment in total) for blood collection. The serum biochemical values from the three fish within each cage were averaged to obtain a single representative value per replicate cage. Thus, the experimental unit for statistical analysis was the net cage (*n* = 3 per treatment). Blood samples (0.8 mL each fish) were obtained from the caudal vein with non-heparinized needles. The blood samples were placed in microcentrifuge tubes and let to clot at room temperature for 4 h prior to serum collection. They were then centrifuged at 5000 rpm for 15 min at 4 °C. The supernatants were meticulously transferred to sterile serum tubes and preserved at −20 °C for a maximum of 7 days before usage. The frozen serum samples were subsequently delivered to the Chiang Mai Veterinary Laboratory Centre Limited Partnership in Chiang Mai, Thailand, for analysis. Serum indices, such as glucose, total protein, albumin, globulin, triglycerides, cholesterol, alanine transaminase (ALT), and aspartate transaminase (AST), were evaluated utilizing an analytical chemistry analyzer (PC400, HORIBA, Japan).

### 2.6. Amino Acid Composition in Fish Muscles

At the end of the 60-day experimental period, fish were sampled from each of the three replicate cages per dietary treatment. From each replicate cage, fillets from both lateral sides of the sampled fish were collected separately to obtain at least 500 g per replicate. Each sample was then analyzed independently for amino acid composition using the method described in Section 2.3. Therefore, the experimental unit for fillet amino acid analysis was the replicate cage (*n* = 3 per treatment). Total essential amino acids (EAA) were calculated as the sum of all detected individual EAA (arginine, histidine, isoleucine, leucine, lysine, phenylalanine, threonine, valine, and methionine) for each replicate sample. Tryptophan was below the limit of detection in all samples and was therefore excluded from the sum. Cystine and hydroxyproline are non-essential amino acids and were not included in the total EAA.

### 2.7. Microbiota Study

#### 2.7.1. Sample Collection and DNA Extraction

At the end of the 60-day feeding trial, intestinal samples were collected from red tilapia fed the four experimental diets (T1–T4) immediately before bacterial challenge and again at 7 days post-challenge. During the 7-day post-challenge period, fish continued to receive their respective experimental diets at the same feeding rate (5% body weight per day) as during the pre-challenge feeding trial. For each dietary treatment and time point, five fish were randomly selected and euthanized using an overdose of MS-222 (tricaine methane sulfonate, 300 mg/L). Post-challenge samples were collected 7 days after intraperitoneal injection with *A. hydrophila* at 10^7^ CFU/mL.

*Aeromonas hydrophila* strain DMST 21250 was obtained from the Department of Medical Sciences, Ministry of Public Health, Thailand. To obtain a fresh, actively growing culture, the strain was cultured aerobically in Tryptic Soy Broth (TSB) at 37 °C for 18–24 h. The bacterial suspension was adjusted with sterile physiological saline (0.85% NaCl) to a turbidity equivalent to 1.0 × 10^7^ CFU/mL, as confirmed by serial dilution and colony enumeration on Tryptic Soy Agar.

Prior to the main experiment, a preliminary dose-finding trial was conducted to determine an appropriate sublethal concentration for red tilapia (*Oreochromis* spp.) of similar size and reared under identical conditions. Graded doses of *A. hydrophila* (10^5^, 10^6^, 10^7^, and 10^8^ CFU/mL) were administered via intraperitoneal injection (0.1 mL per fish), and mortality was monitored for 7 days post-injection. The 10^7^ CFU/mL dose consistently resulted in survival exceeding 95% with no overt clinical signs of disease, whereas higher doses (10^8^ CFU/mL) caused significant mortality. Accordingly, 10^7^ CFU/mL was selected as a sublethal concentration for the main experiment. This exposure provided a defined sampling context for describing pooled intestinal microbiota profiles before and after bacterial administration, as a secondary exploratory component of the feeding study.

At the end of the 60-day feeding trial, fish from each dietary treatment group were randomly selected and administered a single intraperitoneal injection. Each group consisted of 30 fish, divided into three replicates of 10 fish. For the T1 (control, 0% fishmeal replacement) treatment, fish were divided into two subgroups of 30 fish each (three replicates of 10 fish per subgroup). One T1 subgroup (negative control) was injected with 0.1 mL of sterile physiological saline (0.85% NaCl), while the other T1 subgroup (positive control) and the T2, T3, and T4 treatment groups were injected with 0.1 mL of the *A. hydrophila* suspension (10^7^ CFU/mL). All groups were housed under identical conditions and monitored for 7 days post-injection. No mortality was observed in any group during this period. No mortality was observed in any group during this period, confirming the sublethal nature of the challenge. Intestinal samples for microbiota analysis were collected immediately before the challenge (pre-challenge) and at 7 days post-challenge, as described in Section 2.7.1.

The intestinal tract was dissected aseptically. Intestinal contents were gently flushed into sterile tubes using sterile phosphate-buffered saline (PBS). For each treatment and time point, intestinal samples from five fish were pooled prior to DNA extraction, generating one composite microbiome sample per group (*n* = 1 pooled sample per treatment per time point). The pooled composite, rather than each of the five contributing fish, was considered the analytical unit for microbiota profiling. Consequently, the microbiota data were interpreted descriptively and were not subjected to inferential statistical comparisons among treatments or sampling points. This component was explicitly exploration and intended for hypothesis generation.

Microbial community DNA was extracted from the pooled samples using the TGuide S96 Magnetic Universal DNA Kit (DP812, Tiangen Biotech, Beijing, China) following the manufacturer’s instructions. DNA concentration and purity were assessed using a NanoDrop 2000 spectrophotometer (Thermo Fisher Scientific, Wilmington, DE, USA). A negative control sample was included to monitor potential contamination during DNA extraction and sequencing.

#### 2.7.2. 16S rRNA Gene Amplification and Sequencing

The V3–V4 hypervariable region of the bacterial 16S rRNA gene was amplified by polymerase chain reaction (PCR) using the universal primer set 338F (5′-ACTCCTACGGGAGGCAGCA-3′) and 806R (5′-GGACTACHVGGGTWTCTAAT-3′). Both forward and reverse primers were ligated with sample-specific Illumina index sequences to enable multiplexing.

The PCR mixture contained 5 μL of Q5 reaction buffer (5×), 5 μL of Q5 High-Fidelity GC buffer (5×), 0.25 μL of Q5 High-Fidelity DNA Polymerase (5 U/μL), 2 μL of dNTPs (2.5 mM each), 1 μL of each forward and reverse primer (10 μM), 2 μL of DNA template and 8.75 μL of nuclease-free water. The thermal cycling program consisted of initial denaturation at 98 °C for 2 min, followed by 25 cycles of denaturation at 98 °C for 15 s, annealing at 55 °C for 30 s and extension at 72 °C for 30 s, with a final extension at 72 °C for 5 min.

PCR products were assessed for fragment size and integrity using a LabChip GX system (PerkinElmer, Waltham, MA, USA). Qualified libraries were sequenced on an Illumina NovaSeq 6000 platform (Illumina, San Diego, CA, USA) [20] using paired-end 250 bp sequencing. Library construction and sequencing services were provide by Biomarker Technologies (Hong Kong) Co., Ltd, Hong Kong, China.

#### 2.7.3. Bioinformatics Processing

Raw sequencing reads were quality-filtered and trimmed using Trimmomatic V0.33 [21]. Primer sequences were identified and removed using Cutadapt V1.9.1 [22]. The DADA2 pipeline V1.14.1 [23] implemented in RStudio V1.3.1093 [24] was used to denoise high-quality reads, remove chimeric sequences and infer exact amplicon sequence variants (ASVs). Taxonomic assignment of ASVs was performed using the Ribosomal Database Project (RDP) naïve Bayesian classifier trained against the SILVA 16S rRNA reference database V138 [25].

Taxonomic profiles were summarized at the phylum and genus levels. Observed ASV features, ACE, Chao1, Simpson, Shannon, and phylogenetic diversity whole tree were used to describe diversity within each pooled sample, while Good’s coverage characterized sequencing coverage. Bray–Curtis and weighted UniFrac distances summarized dissimilarities between pooled profiles, and UPGMA dendrograms visualized their hierarchical relationships. These analyses and visualizations were performed using QIIME 2 V2020.2 [26] and R packages [24], including vegan and ggplot2. Venn diagrams generated in R [24] described shared and unique ASVs among the compared pools.

These outputs describe different attributes of the same pooled dataset and do not constitute independent evidence of treatment effects. With one pooled sample per treatment and time point, within-treatment biological variation could not be estimated, and no inferential comparisons among dietary treatments or sampling times were performed.

#### 2.7.4. Functional Prediction

Predicted functional profiles of the microbial communities were generated from 16S rRNA ASV data using PICRUSt2 [27]. ASV sequences were placed into a reference phylogenetic tree, and gene family abundances were predicted for each pooled sample. Predicted functional annotations were mapped to Kyoto Encyclopedia of Genes and Genomes (KEGG) Orthologs and summarized at KEGG level 2 pathway categories [28].

Predicted functional categories were compared descriptively between the pre-exposure and post-exposure pools within each dietary treatment. Their purpose was to document predicted functional composition and identify categories for future direct investigation. These outputs represent inferred metabolic potential, not measurements of gene expression, enzymatic activity, or metabolite production, and were not used to explain the measured host outcomes.

### 2.8. Statistical Analysis

Data for growth performance and serum biochemical indices were analysed using cage-level means (*n* = 3 per treatment) to account for the nested structure of the experimental design. All variables were first checked for normality and homogeneity of variance and subsequently analysed using one-way analysis of variance (ANOVA), followed by Tukey’s post hoc test to compare means at a 95% confidence level (*p* < 0.05). For comparisons between *Azolla microphylla* meal (AMM) and commercial fish meal (CFM) presented in Table 3 and Table 4, an independent samples test with Welch’s correction (unequal variances assumed) was applied. Results are expressed as means ± standard deviations. Statistical analyses were performed using STATA version 18. Microbiota diversity indices, taxonomic profiles, ASV distributions, and PICRUSt2-derived functional profiles were excluded from inferential statistical analyses because each treatment-by-time point was represented by one pooled composite sample.

## 3. Results

### 3.1. Amino Acid and Fatty Acid Profiles of Azolla microphylla Meal (AMM), Commercial Fish Meal (CFM)

Amino acids were significantly lower in AMM than in commercial fish meal (*p* < 0.001) across both essential and non-essential categories. Total essential amino acids were 5058 mg/100 g in Azolla versus 19,650 mg/100 g in fish meal, and total non-essential amino acids were 7003 versus 29,836 mg/100 g, respectively. Methionine, cystine, and hydroxyproline were not detected in Azolla (below LOD) but were present in fish meal. Notably, fish meal exhibited markedly higher lysine, arginine, leucine, glycine, and glutamic acid. Statistical comparisons were conducted using independent *t*-tests with Welch’s correction (Table 3).

Saturated fatty acids were undetectable in AMM, while CFM exhibited a broad SFA profile (notably palmitic and stearic acids). Among MUFA, only cis-9-oleic acid was present in Azolla (0.56 g/100 g), resulting in lower total MUFA than fish meal (0.56 vs. 3.05 g/100 g; *p* < 0.05). For PUFA, Azolla contained cis-11,14-eicosadienoic acid (C20:2) at a markedly higher level than fish meal (1.57 vs. 0.04 g/100 g; *p* < 0.001), whereas other n-3 and n-6 species (including EPA and DHA) were detected only in fish meal. Consequently, total unsaturated fat was lower in Azolla than in fish meal (2.13 vs. 4.82 g/100 g; *p* < 0.01). Omega-3 and omega-6 were undetectable in Azolla but present in fish meal (816.5 and 872.2 mg/100 g), and omega-9 was substantially lower in Azolla (563.4 vs. 1990.8 mg/100 g; *p* < 0.001). All non-detected values were below the LOD (0.01 g/100 g) (Table 4).

### 3.2. Amino Acid and Fatty Acid Profiles of the Four Experimental Diets

Consistent decline in most amino acids from T1 to T4. Among EAA, arginine, histidine, isoleucine, leucine, lysine (*p* < 0.001), phenylalanine, and valine decreased significantly; threonine was not significant (*p* = 0.055). Methionine was lower in T3 and fell below LOD in T4; tryptophan was detectable in T1–T2 but <LOD in T3–T4. Consequently, ΣEAA declined across diets, with sums for T3–T4 censored by LOD. For NEAA, alanine, aspartic acid, glutamic acid, glycine (*p* < 0.001), proline, and serine decreased stepwise; tyrosine did not differ (*p* = 0.122). Hydroxyproline declined and was ND in T4; cystine approached LOD in T3–T4. ΣNEAA decreased across diets, with T2–T4 affected by values < LOD. Superscript letters denote pairwise differences as indicated (Table 5).

Stepwise reductions in most fatty acids with increasing fishmeal replacement levels. Total SFA decreased from 2.43 to 1.76 g/100 g (*p* < 0.001), driven by declines in myristic, palmitic, heptadecanoic, and stearic acids; lauric became ND at T4. Total MUFA fell from 1.87 to 1.36 g/100 g (*p* = 0.022), with decreases in palmitoleic, eicosanoid, and nervonic acids, while oleic acid was unchanged (*p* = 0.107). Total PUFA declined from 1.30 to 0.65 g/100 g (*p* = 0.011); linoleic acid did not differ (*p* = 0.356), but γ-linolenic, α-linolenic, C20:3n6, EPA, and DHA decreased, with EPA ND at T4 and DHA dropping from 0.09 to 0.01 g/100 g (*p* < 0.001). Consequently, total unsaturated fat decreased (3.03 to 2.03 g/100 g; *p* < 0.001). Omega-3, omega-6, and omega-9 contents declined progressively (*p* < 0.001, 0.001, and 0.048, respectively). Trans fat was low or ND across diets. Superscript letters indicate pairwise differences among treatments (Table 6).

### 3.3. Growth Performance and Survival

**Figure 1:**
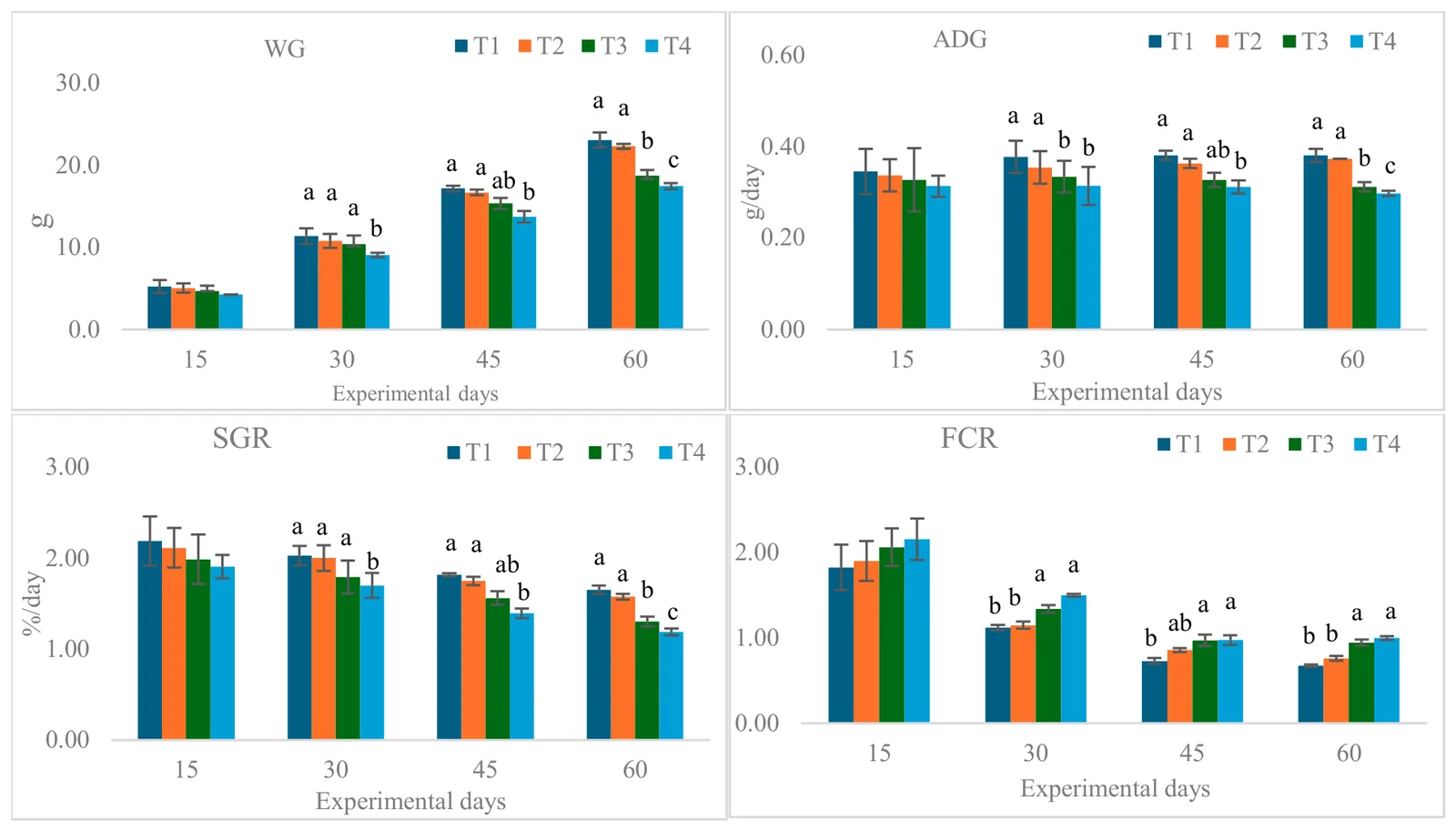
Growth performance of red tilapia (*Oreochromis* spp.) fed diets in which fish meal was replaced with different levels of *Azolla microphylla* meal for 60 days. WG, weight gain; ADG, average daily gain; SGR, specific growth rate; FCR, feed conversion ratio. Different letters indicate significant differences (*p* < 0.05).

A clear dose–response to increasing fishmeal replacement. WG, ADG, and SGR declined stepwise across treatments, with the lowest replacement (T1) exhibiting the highest values and the highest replacement the lowest. Correspondingly, FCR increased progressively, indicating reduced feed efficiency at greater fishmeal replacement. Letter groupings denote pairwise differences (*p* < 0.05): the highest replacement group differed from T1 and typically from intermediate levels, whereas adjacent groups that share letters did not differ. Overall, performance deterioration was monotonic, with the most pronounced reductions at the highest fishmeal replacement level (Figure 1).

### 3.4. Serum Biochemistry

**Figure 2:**
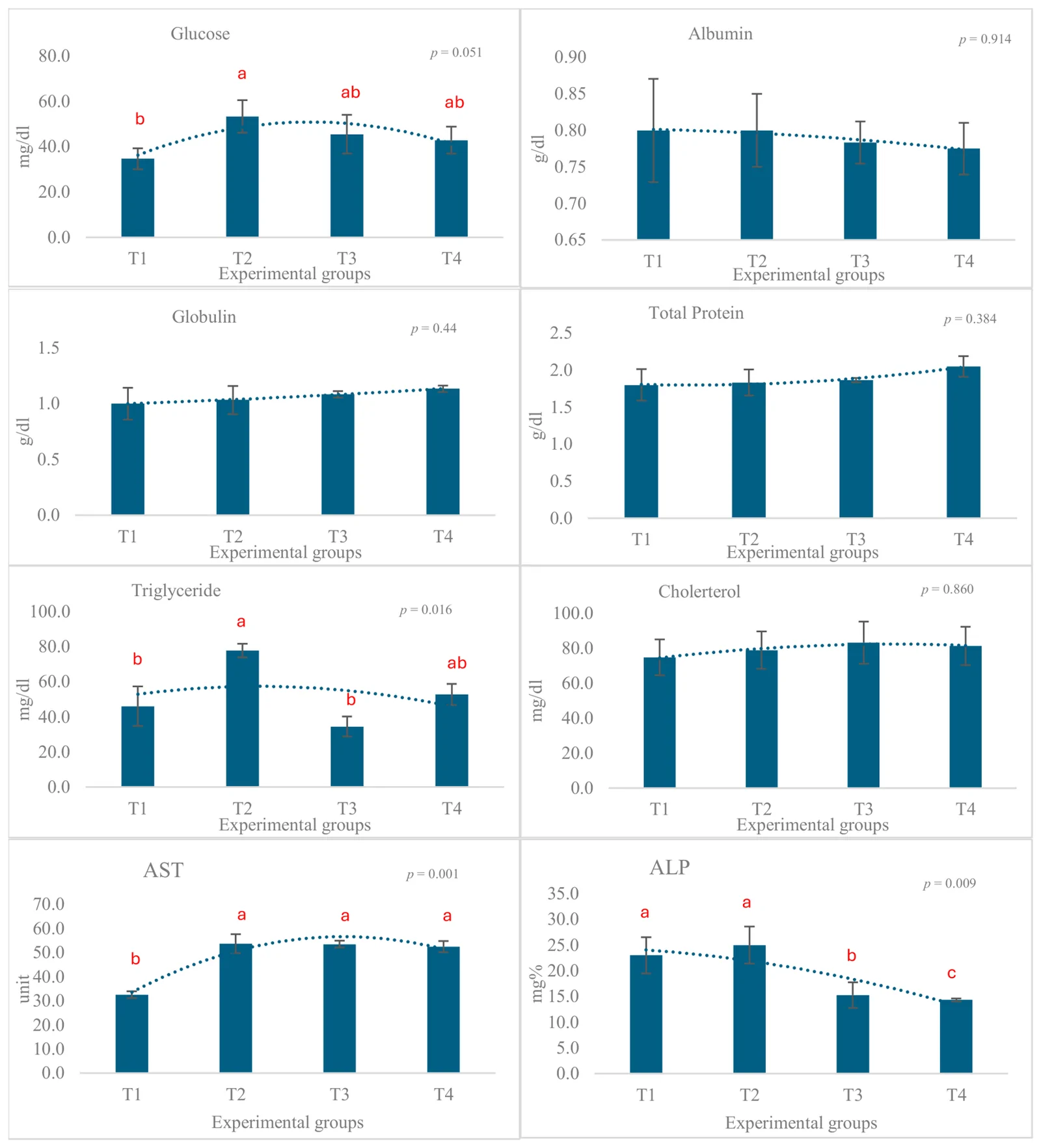
Biochemical indices of red tilapia (*Oreochromis* spp.) fed diets in which fish meal was replaced with different levels of *Azolla microphylla* meal for 60 days. AST, aspartate transaminase; ALP, alkaline phosphatase. Different letters indicate significant differences (*p* < 0.05).

Serum biochemical parameters were significantly influenced by the experimental diets (Figure 2). Blood glucose levels showed a fluctuating pattern across treatments, with the highest value observed in T2 and the lowest in T1; no clear dose-dependent trend was observed for this parameter. Albumin, globulin, total protein and Serum cholesterol exhibited were not affected by fish meal replacement by *A. microphylla*. Triglyceride levels differed significantly (*p* < 0.05); however, this change exhibited a fluctuating pattern, with the highest value observed in T2 and the lowest in T3. In contrast, AST and ALP exhibited opposite patterns of response: AST increased with higher levels of fishmeal replacement by Azolla meal, whereas ALP showed an inverse trend.

### 3.5. Amino Acid and Fatty Acid Profiles of Fillets from the Four Treatment Groups

Muscle amino acid profiles were largely conserved across diets. Individual EAA and NEAA did not differ significantly among treatments (*p* > 0.05); tryptophan was below LOD in all groups, and cystine and hydroxyproline were not detected. Despite this stability at the component level, total EAA (calculated as the sum of all detected individual EAA) was significantly lower in T4 than in T1–T3 (7535 vs. 8148–8203 mg/100 g; *p* = 0.031), representing an ~8% reduction at the highest fishmeal replacement level (Table 7). This likely reflects the cumulative effect of small, consistent numerical reductions in multiple EAA that individually did not reach statistical significance. Total NEAA showed a nonsignificant downward trend in T4 (8440 vs. ~9048–9157 mg/100 g; *p* = 0.054). These results suggest that high fishmeal replacement modestly decreases summed EAA in muscle without altering most individual amino acids detectably.

### 3.6. Survival Following Low-Dose A. hydrophila Challenge

At 7 days post-challenge, survival rates ranged from 95.6% to 98.9%, with no significant differences among dietary treatments (one-way ANOVA, *p* > 0.05). The bacterial exposure was therefore sublethal under the conditions tested rather than a lethal disease challenge. Because immune parameters were not measured, the exposure cannot be interpreted as confirmed Immunostimulantion. The post-challenge microbiota profiles are consequently presented only as exploratory observations following sublethal *A. hydrophila* exposure.

### 3.7. Exploratory Descriptive Profiles of Intestinal Microbiota Before and After Bacterial Exposure

For each treatment-by-time point, intestinal microbiota profiling was conducted using one pooled sample comprising five fish (*n* = 1 pooled sample). Sample suffixes B and F indicate sampling immediately before bacterial exposure and at 7 days post-exposure, respectively. Good’s coverage was at least 0.9999 for all pooled intestinal samples. The negative control sample (Neg-Ctr), derived from T1 fish injected with sterile saline, was included in Table 8 solely as a descriptive saline-reference profile and was not used to infer dietary or bacterial-exposure effects. Accordingly, all values, numerical contrasts, and visual patterns reported below characterize only the sequenced pools; they do not estimate treatment effects or variability among individual fish.

Alpha diversity: At the pre-exposure sampling point, the T2B pooled profile contained 972 observed features and had a Shannon index of 8.138, while the observed feature counts for T1B, T3B, and T4B ranged from 47 to 79 (Table 8). The observed feature counts for T2B and T2F were 972 and 40, respectively, and those for T4B and T4F were 79 and 615, respectively. The Shannon indices recorded for T2F and T4F were 2.400 and 5.882, respectively. These values are reported as characteristics of the individual sequenced pools and were not subjected to inferential statistical comparisons across treatments or sampling points.

**Figure 3:**
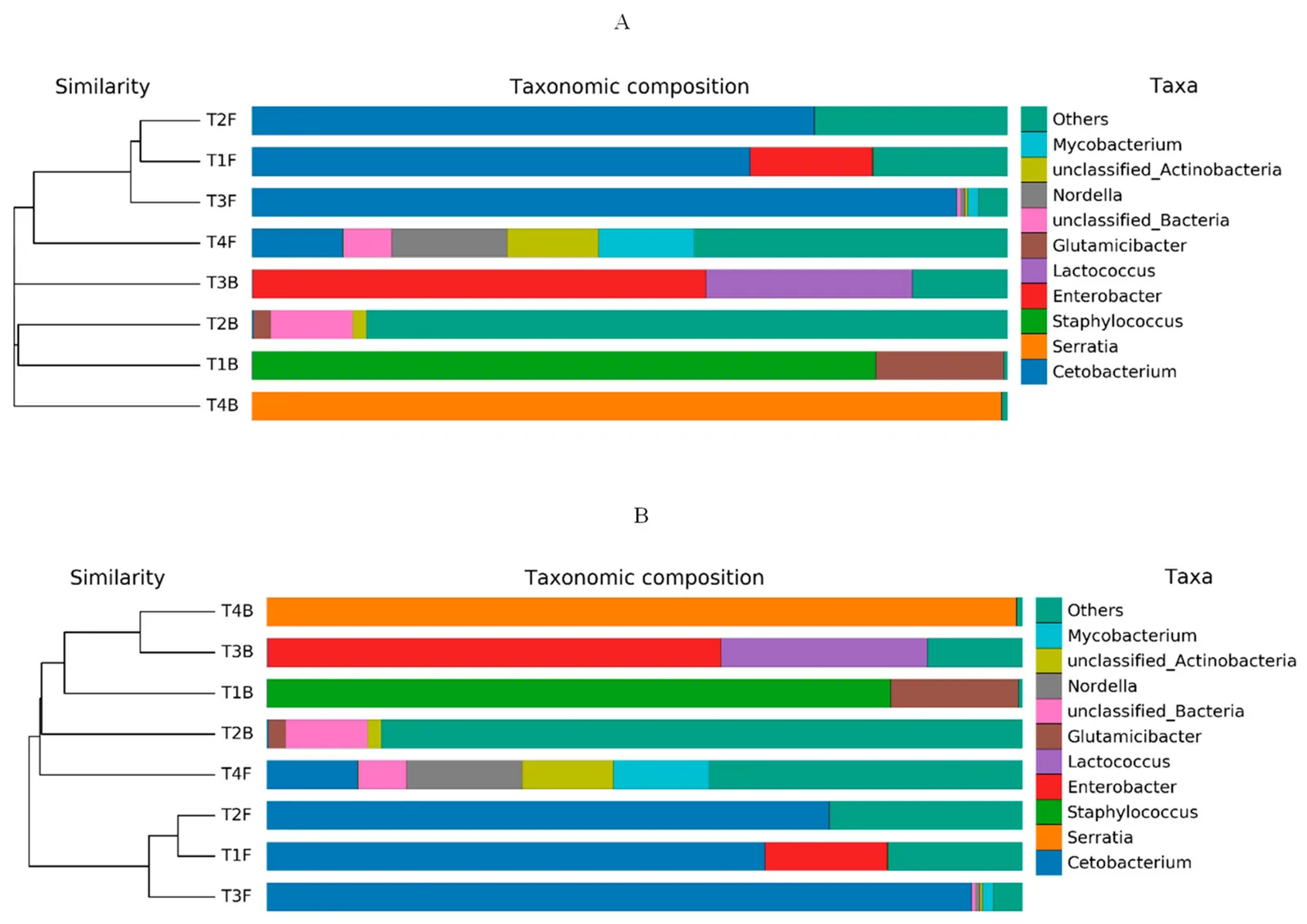
UPGMA clustering dendrograms of intestinal microbial communities based on (**A**) Bray–Curtis and (**B**) weighted UniFrac distances. The dendrograms show the hierarchical clustering of gut microbiota profiles from red tilapia (*Oreochromis* spp.) across four dietary treatments (T1–T4). T1–T4 represent diets with 0%, 25%, 50%, and 75% fishmeal replacement, respectively. Increasing AMM inclusion was accompanied by increasing soybean meal levels and other changes in diet formulation (Table 1). “Pre” indicates samples collected immediately before intraperitoneal injection of *Aeromonas hydrophila* (10^7^ CFU/ml), and “Post” indicates samples collected 7 days post-challenge. The bar plots below each dendrogram depict the relative abundance of dominant bacterial genera, illustrating genus-level compositional differences. The dendrograms provide descriptive visualizations of distances among the pooled profiles. Each treatment-by-time point was represented by one pooled composite sample; therefore, the clustering does not constitute statistical evidence of community convergence, treatment separation, or dietary effects.

**Figure 4:**
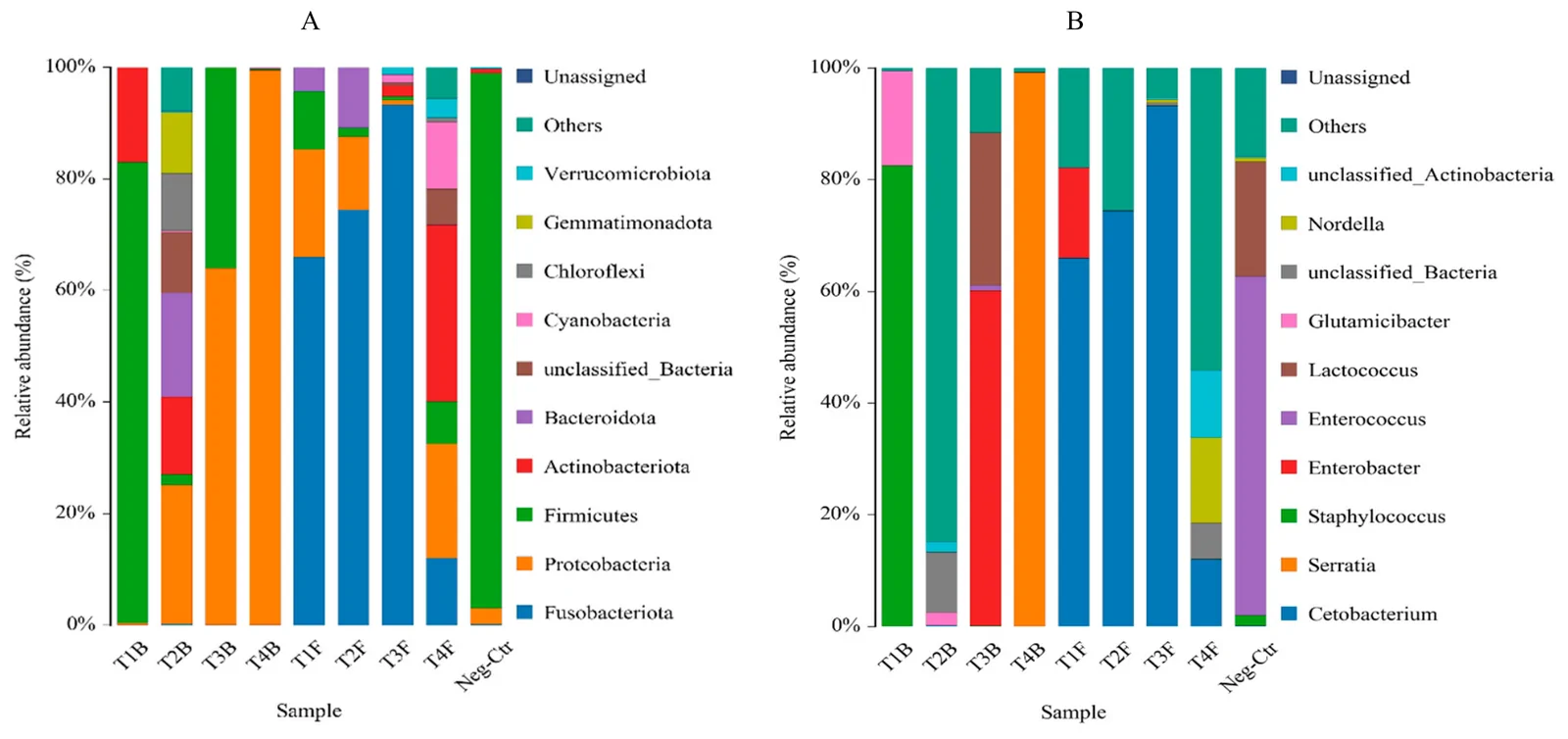
Relative abundance of dominant bacterial phyla (**A**) and genera (**B**) detected in pooled intestinal microbiota samples. T1–T4 represent diets with 0%, 25%, 50%, and 75% fishmeal replacement, respectively. Increasing AMM inclusion was accompanied by increasing soybean meal levels and other changes in diet formulation (Table 1). Pre and Post indicate samples collected before and 7 days after the sublethal *A. hydrophila* exposure. Each bar represents one pooled composite sample containing intestinal material from five fish. Taxa with low relative abundance were grouped as “Others.” The profiles are presented descriptively without statistical inference.

Beta diversity and taxonomy: UPGMA dendrograms based on Bray–Curtis and weighted UniFrac distances summarized dissimilarities among the pooled profiles (Figure 3). T1F, T2F, and T3F were positioned more closely in the dendrograms, whereas T4F was positioned separately. *Cetobacterium* represented a high relative proportion in T1F–T3F, particularly T3F, while T4F contained a mixture of *Cetobacterium, Nordella*, unclassified Actinobacteria, and other genera (Figure 4).

**Figure 5:**
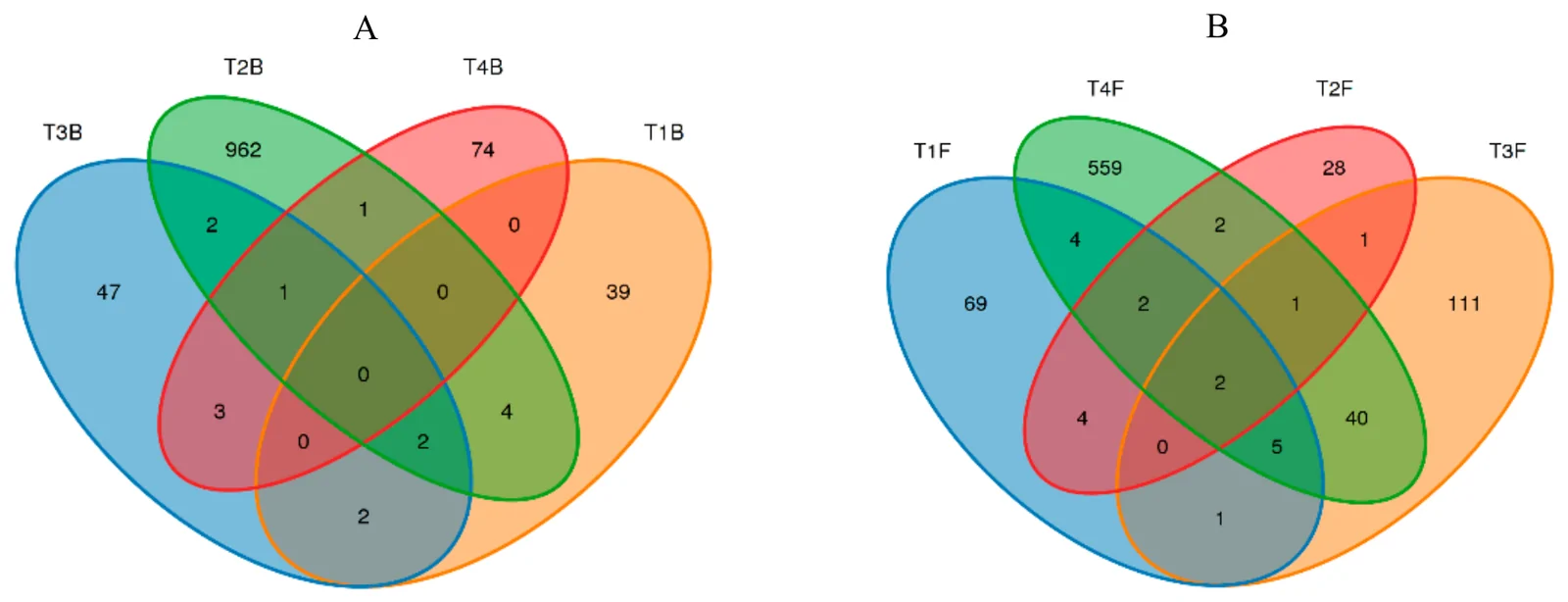
Distribution of amplicon sequence variants (ASVs) among pooled intestinal microbiota profiles based on pooled intestinal samples (exploratory, descriptive data). Venn diagrams display the number of shared and unique ASVs in the intestinal microbiota of red tilapia. (**A**) Pre-challenge samples; (**B**) Post-challenge samples (7 days after *A. hydrophila* injection). As each treatment is represented by one composite sample (*n* = 1), these Venn distributions serve to illustrate ASV overlap within the sequenced pools and are presented as hypothesis-generating observations rather than statistically inferred community differences. Numbers in overlapping regions indicate ASVs shared between or among the respective treatments.

Shared and unique ASV distributions: The Venn diagrams show ASV overlap among the sequenced pools (Figure 5). Before exposure, T2B contained 962 unique features, compared with 39–74 in the other pre-exposure pools. After exposure, T4F contained the largest number of unique features (559), while T2F contained 28. Two ASVs were shared across all four post-exposure pools. Here, unique and shared features refer only to their occurrence within the compared pools.

**Figure 6:**
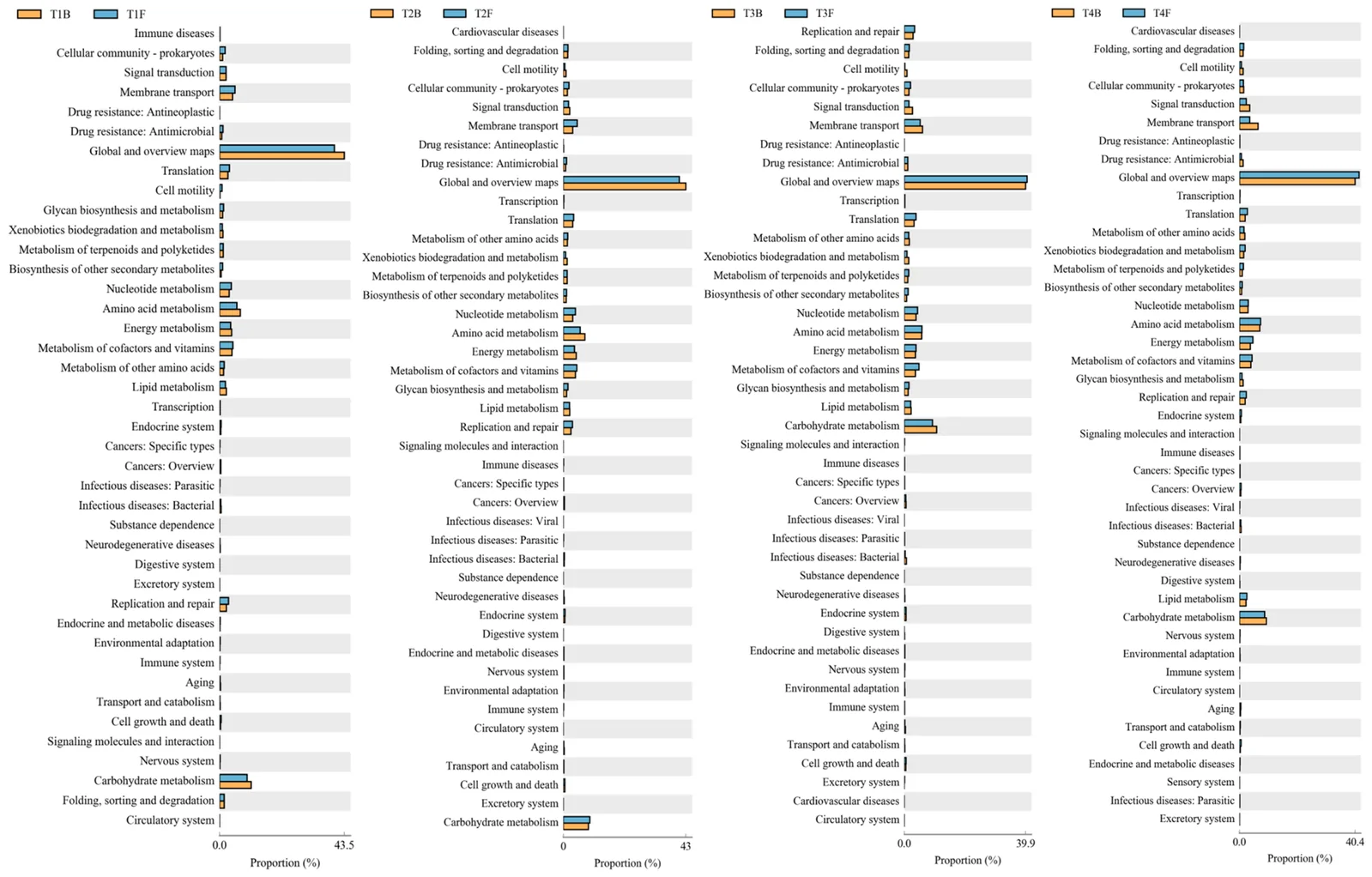
Descriptive PICRUSt2-predicted functional profiles of pooled intestinal microbiota samples based on KEGG Level 2 classifications. Panels A–D show the relative proportions of predicted functional categories in the pooled samples collected before (B) and after (F) sublethal *Aeromonas hydrophila* exposure for T1, T2, T3, and T4, respectively. T1–T4 represent diets with 0%, 25%, 50%, and 75% fishmeal replacement, respectively. Increasing AMM inclusion was accompanied by increasing soybean meal levels and other changes in diet formulation (Table 1). Each treatment-by-time point was represented by one pooled composite sample containing intestinal material from five fish. Therefore, the profiles are presented descriptively, and no inferential statistical comparisons were performed. The predicted categories represent inferred metabolic potential rather than direct measurements of gene expression, enzymatic activity, or metabolite production.

Predicted functions: PICRUSt2 profiles were dominated by KEGG Level 2 categories comprising global and overview maps, carbohydrate metabolism, amino acid metabolism, energy metabolism, metabolism of cofactors and vitamins, membrane transport, translation, and nucleotide metabolism (Figure 6). Relative to T1B, T1F had numerically lower predicted proportions of global and overview maps, carbohydrate metabolism, and amino acid metabolism, and higher proportions of membrane transport, translation, nucleotide metabolism, and replication and repair. Relative to T2B, T2F had lower proportions of global and overview maps and amino acid metabolism, and slightly higher proportions of carbohydrate metabolism, membrane transport, translation, nucleotide metabolism, and metabolism of cofactors and vitamins.

The T3 profiles were broadly similar for several major categories; relative to T3B, T3F had higher predicted proportions of translation, nucleotide metabolism, replication and repair, and amino acid metabolism, and a lower proportion of carbohydrate metabolism. Relative to T4B, T4F had higher proportions of global and overview maps, amino acid metabolism, energy metabolism, metabolism of cofactors and vitamins, and translation, and lower proportions of carbohydrate metabolism, membrane transport, and signal transduction. These are numerical descriptions of predicted profiles, not confirmed functional responses.

## 4. Discussion

The pronounced differences in both amino acid and fatty acid profiles between AMM and CFM underscore the nutritional limitations of AMM as a standalone feed ingredient, particularly with respect to essential amino acids and long-chain polyunsaturated fatty acids [29]. The absence of methionine and the markedly lower concentrations of other essential amino acids in AMM indicate that supplementation would be required to meet the dietary requirements of most aquaculture species [30], which is critical because essential amino acid profiles directly influence growth performance [13]. Although AMM contains relatively high proportions of mono- and polyunsaturated fatty acids, the lack of key omega-3 and omega-6 fatty acids such as EPA and DHA further reduces its nutritional completeness for aquatic animals [31]. Nevertheless, aquatic macrophytes, including Azolla, are recognized as rich, low-cost sources of fatty acids, proteins and minerals, and thus remain promising candidates for aquafeeds, especially given their high productivity and availability [32]. To exploit this potential, however, strategic fortification or co-formulation with complementary protein and lipid sources is necessary to achieve nutritionally balanced diets [33]. Indeed, previous studies have shown that macrophytes such as *A. pinnata* can replace fishmeal at inclusion levels up to about 10% without impairing growth or nutrient utilization, particularly when crude protein content is high (up to ~28%) and amino acid profiles are favorable [32,34]. By contrast, the comparatively lower amino acid and fatty acid quality observed for *A. microphylla* in the present study [31,34] highlights that not all Azolla species are nutritionally equivalent.

Fishmeal was replaced with a combination of AMM and soybean meal to maintain isonitrogenous and isoenergetic diets, a common approach in fishmeal-replacement studies. This design introduces confounding, so effects on growth, serum biochemistry, and fillet composition cannot be ascribed solely to AMM. Because soybean meal is methionine-limiting, it may have contributed to amino acid imbalance across treatments. Although this strategy reflects practical formulation, it obscures AMM’s specific role. Future work should isolate AMM’s effects by holding soybean meal constant or using AMM alone with crystalline amino acid supplementation.

The observed disparity in amino acid concentrations highlights a significant nutritional bottleneck in *A. microphylla* when compared to the balanced profile of fish meal, which is essential for optimizing protein synthesis in *Oreochromis* species [35]. Specifically, the complete absence of sulfur-containing amino acids such as methionine and cystine in *A. microphylla* underscores the necessity for strategic supplementation or synthetic amino acid fortification to prevent growth retardation in tilapia [7]. This deficiency in limiting amino acids, coupled with lower levels of lysine and arginine in the plant-based meal, likely explains the declining growth performance and feed efficiency observed as fishmeal replacement levels increased [36,37]. Furthermore, these amino acid constraints align with previous meta-analyses indicating that plant-based protein sources frequently lack the precise essential amino acid density required to match the nutritional profile of high-quality fishmeal [10]. Such compositional disparities, particularly the reduced bioavailability of essential amino acids in alternative ingredients, often necessitate precise crystalline amino acid balancing to achieve growth rates comparable to those observed in fishmeal-based diets [38,39].

This striking fatty acid divergence, characterized by the absence of critical long-chain polyunsaturated fatty acids such as DHA in the fern, reflects the inherent lipid profile limitations that constrain Azolla as a total fishmeal replacer [40]. Consequently, the reliance on *A. microphylla* necessitates careful consideration of these lipidic deficits, as the lack of essential long-chain fatty acids may compromise both the physiological health of the fish and the nutritional quality of the final product for human consumption [41]. While previous studies on alternative macrophytes like *Lemna minor* have demonstrated potential for enhancing long-chain polyunsaturated fatty acid content, the distinct biochemical profile of *A. microphylla* necessitates further investigation into whether lipid supplementation can mitigate these identified nutritional gaps [42]. Furthermore, the presence of anti-nutritional factors in aquatic ferns, which can bind to essential nutrients and reduce their intestinal bioavailability, may exacerbate these inherent amino acid and fatty acid deficiencies.

This decline in growth metrics is likely attributed to a combination of elevated dietary fiber, high lignin content, the presence of anti-nutritional factors that impede nutrient digestibility and increase energy expenditure, and the simultaneous increase in soybean meal, which may have contributed to amino acid imbalances [43]. Moreover, the observed growth inhibition corroborates findings in other teleosts, where dietary imbalances in essential amino acids or the presence of anti-nutritional factors restrict the utilization of nitrogenous compounds for muscle accretion [13,44]. While the integration of Azolla into diets offers a cost-effective strategy to reduce reliance on expensive marine proteins, the persistent trade-off between economic savings and physiological productivity remains a critical challenge in intensive aquaculture systems [32,45]. Thus, optimizing *A. microphylla* inclusion requires a nuanced approach, likely to involve dietary additives or processing techniques to neutralize metabolic inhibitors and bridge the nutritional gap between plant-derived proteins and the demanding physiological requirements of *Oreochromis* species [46].

Some biochemical parameters were affected by fish meals being replaced with *A. microphylla* meal. This indicates that while some metabolic pathways may be altered, the overall impact on key indicators of liver function, such as AST and ALP, suggests a complex physiological response to varying levels of fishmeal replacement with AMM-containing diets [47]. Specifically, elevated AST levels, as observed in some studies utilizing Azolla meal, can indicate potential hepatic stress or cellular damage, while fluctuating ALP levels may reflect changes in bone metabolism or hepatobiliary function [48]. Therefore, it is crucial to consider these fluctuating enzyme activities in conjunction with other biochemical markers to comprehensively assess the physiological impact of Azolla inclusion in aquatic diets [49]. This necessitates further investigation into the specific mechanisms underlying these enzymatic shifts, particularly concerning how Azolla’s unique phytochemical composition might interact with metabolic pathways to elicit such responses [29]. For instance, previous research has indicated that increased dietary Azolla can lead to elevated AST and ALP levels, suggesting a burden on liver metabolism in fish [47]. However, other studies have found that while alanine aminotransferase and aspartate aminotransferase activities were higher in fish fed Azolla, these values remained within normal physiological ranges, suggesting adaptive responses rather than overt pathological damage [50]. This divergence in findings highlights the importance of species-specific metabolic responses and the varied processing methods of Azolla meal, which can influence nutrient bioavailability and the presence of antinutritional factors [47]. Further research should therefore focus on optimizing Azolla processing techniques to mitigate potential anti-nutritional effects and enhance nutrient digestibility, thereby improving its efficacy as a sustainable aquaculture feed ingredient [11].

This reduction in total essential amino acids is consistent with observations in other teleosts, where reliance on plant-based proteins often leads to an inferior nutrient composition compared to traditional fishmeal-based diets [36]. Such amino acid imbalances are frequently mitigated in other aquaculture models through the targeted supplementation of crystalline amino acids, which effectively restores growth performance and metabolic stability to levels comparable to high-fishmeal diets [51]. However, the efficacy of this supplementation strategy remains complex, as evidence indicates that rapid uptake of crystalline amino acids can create plasma imbalances when compared to the steady absorption rates inherent in whole-protein fishmeal [52]. In addition to amino acid modulation, the substitution of fishmeal with Azolla significantly alters the fatty acid composition of muscle tissue, as plant-derived profiles often lack the long-chain polyunsaturated fatty acids essential for optimal teleost health [53].

Exploratory profiling revealed sample-level differences in diversity and taxonomic composition. *Cetobacterium* represented a high relative proportion in the pooled post-exposure profiles of T1–T3, whereas T4F contained a broader mixture of detected genera. *Cetobacterium* is commonly detected in freshwater fish and has been associated with vitamin B12 production, acetate generation, and carbohydrate fermentation [54,55]. These published associations provide biological context for the detected genus, but the corresponding activities were not measured in the present samples. Neither its relative abundance nor the PICRUSt2 predictions establish a microbial contribution to the growth, serum biochemical, or fillet amino acid outcomes.

The microbiota findings describe pooled communities from fish receiving different dietary formulations containing increasing levels of AMM. Soybean meal increased alongside AMM, and other formulation components also varied (Table 1); the observed profiles therefore cannot be attributed exclusively to *A. microphylla*. Replicated research has shown that plant-based diets can influence fish intestinal microbiota [56]; however, the present pooled dataset does not establish effects of either AMM or the dietary formulations as a whole. These observations provide hypotheses for future studies incorporating independent biological replication, measurements of microbial metabolites, and functional intestinal indicators. Such studies should distinguish the contribution of AMM from accompanying dietary changes and directly evaluate predicted microbial functions.

Azolla could also contribute value beyond protein supply through local cultivation, partial reduction of feed costs, and integration with resource-recovery or low-input aquaculture systems, although these benefits require confirmation through ingredient-specific economic, environmental, and nutritional assessments [40,57]. Furthermore, aligning such nutritional strategies with the ecological intensification of small-scale aquaculture like traditional practices involving floating macrophytes may provide a viable pathway for reducing reliance on commercial pellets without compromising long-term stock productivity [14].

Limitations of the study: Several limitations of this study should be acknowledged to facilitate appropriate interpretation of the findings. First, and most importantly, the intestinal microbiota analysis used one pooled sample per treatment and time point because of resource constraints. This sampling design did not permit estimation of between-fish and between-cage variation within each treatment and time point, and the five contributing fish could not be treated as independent microbiota replicates. The analyses therefore describe the sampled pools without establishing dietary or exposure effects. The different analytical outputs derive from the same dataset and do not overcome this limitation.

Second, post-challenge immune parameters (e.g., lysozyme, complement activity, cytokine expression) were not measured, which limits our ability to directly link the descriptive microbial shifts to host immune status or disease resistance.

Third, the sublethal challenge dose resulted in >95% survival across all groups, confirming that the exposure was sublethal under the conditions tested. However, because immune parameters were not measured, Immunostimulantion cannot be confirmed.

Fourth, the experiment was conducted under controlled net-cage conditions in an earthen pond, which may not fully reflect the variable water quality, stocking densities, and management practices encountered in large-scale commercial aquaculture systems.

Fifth, fishmeal was replaced by a combination of AMM and soybean meal, so the specific contribution of AMM cannot be isolated from that of soybean meal. This confounding factor should be considered when interpreting the results. Future studies should separate the effects of AMM from those of other dietary ingredients, for example, by keeping soybean meal constant while varying AMM, or by using AMM as the sole fishmeal replacer with amino acid supplementation.

Finally, short-chain fatty acids, vitamin B12, and other microbial metabolites were not measured. Consequently, the PICRUSt2 predictions and published functions associated with detected taxa cannot be taken as evidence of microbial activity in these fish. Future studies should combine independent microbiota replication with direct functional measurements.

## 5. Conclusions

In conclusion, replacing fishmeal with a blend of *A. microphylla* meal (AMM) and soybean meal in red tilapia diets progressively reduced growth performance and feed efficiency, with adverse effects becoming evident above 25% replacement. Serum AST increased and ALP decreased at higher replacement levels. Fillet total essential amino acids (EAA) were maintained up to 50% replacement and declined only at 75%. Accordingly, the optimal replacement level depends on production goals: not exceeding 25% when maximizing growth performance, and up to 50% when prioritizing fillet EAA quality, albeit with reduced growth and increased feed conversion ratio (FCR). Because fishmeal was replaced by AMM plus soybean meal, these effects cannot be attributed exclusively to AMM. The pooled microbiota data are descriptive only; future studies should use replicated designs with amino acid supplementation to isolate AMM-specific effects.

## Figures and Tables

**Table 1:** Ingredients and proximate composition of the four experimental diets.

Ingredient Compositions (%)	Experimental Diets (Dry Weight)			
	0%	25%	50%	75%
Commercial fish meal	25.5	19.13	12.75	6.38
*Azolla microphylla*	0	6.38	12.75	19.13
Soybean meal	25.5	30.49	34.5	39.49
Corn starch	16	13	12	10
Rice bran	13	13	11	11
Broken rice	13	11	9	6
Plant oil	2	2	2	2
Premix	5	5	5	5
Total	100	100	100	100
Proximate composition (% in dry weight)				
Protein	26.89 ± 0.65	26.84 ± 0.92	26.68 ± 0.49	26.58 ± 0.81
Lipid	5.36 ± 0.13	5.33 ± 0.24	5.30 ± 0.34	5.28 ± 0.26
Moisture	10.4 ± 0.14	11.32 ± 0.29	12.25 ± 0.31	12.63 ± 0.33
Ash	13.41 ± 0.41	12.39 ± 0.46	12.12 ± 0.35	11.53 ± 0.38
Fiber	10.85 ± 0.42	11.37 ± 0.46	11.42 ± 0.47	12.53 ± 0.36

Note 1: Premix 1 kg contained vitamins, including vitamin A 500,000 IU, vitamin D3 100,000 IU, vitamin E 10,000 mg, vitamin K 2000 mg, vitamin B1 2500 mg, vitamin B2 1000 mg, vitamin B6 700 mg, vitamin B12 10 mg, vitamin C 10,000 mg, choline chloride 10,000 mg, nicotinamide 3000 mg, pantothenic acid 3000 mg, folic acid 300 mg, Inositol 10,000 mg, biotin 20 mg and media filled to 1000 mg. Note 2: Fishmeal replacement levels were 0%, 25%, 50%, and 75% for T1–T4, respectively. The corresponding dietary AMM inclusion levels were 0%, 6.38%, 12.75%, and 19.13%. Fishmeal was replaced by a combination of AMM and soybean meal. As fishmeal decreased, both AMM and soybean meal increased proportionally to maintain isonitrogenous and isoenergetic diets.

**Table 2:** Proximate compositions of commercial fish meal and *Azolla microphylla* meal.

Ingredients	Protein	Lipid	Moisture	Ash	Fiber
Commercial fish meal	57.03 ± 3.21	10.58 ± 1.52	7.68 ± 0.09	23.53 ± 2.36	0.20 ± 0.02
*Azolla microphylla* meal	24.09 ± 1.26	12.32 ± 0.78	18.85 ± 1.56	19.98 ± 2.69	15.71 ± 2.34

Note: Crude lipid was determined by Soxhlet extraction and represents total extractable lipids, including phospholipids, glycolipids, sterols, waxes, pigments, and other non-saponifiable lipids. Values are expressed as mean ± SD.

**Table 3:** Amino acid profiles of *Azolla microphylla* meal and commercial fish meal.

	Ingredients (mg/100 g)		*p*-Values
Amino Acid Compositions	*Azolla microphylla* Meal	Commercial Fish Meal	
Essential amino acids (EAA)			
Arginine	716.94 ± 20.3	3339.87 ± 52.4	<0.001
Histidine	240.05 ± 15.9	1057.88 ± 82.3	<0.001
Isoleucine	606.08 ± 62.3	1693.09 ± 85.6	<0.001
Leucine	1236.65 ± 95.2	3106.58 ± 110.2	<0.001
Lysine	488.16 ± 98.1	3174.49 ± 156.4	<0.001
Phenylalanine	762.39 ± 69.1	1808.95 ± 153.7	<0.001
Threonine	730.22 ± 75.9	1990.85 ± 188.6	<0.001
Tryptophan	171.68 ± 28.2	329.75 ± 42.8	<0.001
Valine	836.21 ± 41.2	2094.85 ± 98.7	<0.001
Methionine	ND	1053.19 ± 97.5	-
ΣEAA	5058.16 ± 124.7	19,649.5 ± 182.6	<0.001
Non-essential amino acids (NEAA)			
Alanine	915.65 ± 59.1	3706.43 ± 154.3	<0.001
Aspartic acid	1590.24 ± 87.2	4671.11 ± 174.3	<0.001
Glutamic acid	1720.66 ± 65.2	6932.12 ± 128.3	<0.001
Glycine	824.66 ± 78.2	5767.45 ± 167.2	<0.001
Hydroxyproline	ND	1878.67 ± 127.9	-
Proline	646.72 ± 86.9	3141.13 ± 164.7	<0.001
Serine	750.81 ± 98.2	2057.31 ± 130.4	<0.001
Tyrosine	554.05 ± 96.1	1320.35 ± 153.7	<0.001
Cystine	ND	361.05 ± 32.1	-
ΣNEAA	7002.79 ± 136.7	29,835.62 ± 362.7	<0.001

Notes: ND = not detected (below the limit of detection, LOD). The ΣEAA value for *Azolla microphylla* meal is the sum of the detected essential amino acids only; methionine (ND) was excluded from the total because it could not be quantified. LOD: cystine and methionine 100.00 mg/100 g; hydroxyproline 200.00 mg/100 g. *p*-values have been performed by independent *t*-test (Welch’s correction).

**Table 4:** Fatty acid profiles of *Azolla microphylla* meal and commercial fish meal.

	Ingredients (g/100 g)		*p*-Values
Fatty Acid Compositions	*Azolla microphylla* Meal	Commercial Fish Meal	
Lauric acid (C12:0)	ND	0.07 ± 0.002	-
Myristic acid (C14:0)	ND	0.60 ± 0.030	-
Pentadecanoic acid (C15:0)	ND	0.17 ± 0.020	-
Palmitic acid (C16:0)	ND	3.50 ± 0.090	-
Heptadecanoic acid (C17:0)	ND	0.26 ± 0.070	-
Stearic acid (C18:0)	ND	1.38 ± 0.040	-
Arachidic acid (C20:0)	ND	0.03 ± 0.002	-
Saturate fat	ND	6.08 ± 1.670	-
Palmitoleic acid (C16:1n7)	ND	0.70 ± 0.030	-
Cis-9-Oleic acid (C18:1n9c)	0.56 ± 0.020	1.87 ± 0.060	<0.001
Cis-10-Heptadecenoic acid (C17:1n10)	ND	0.03 ± 0.001	-
Trans-9-Elaidic acid (C18:1n9t)	ND	0.10 ± 0.002	-
Cis-11-Eicosenoic acid (C20:1n11)	ND	0.22 ± 0.003	-
Erucic acid (C22:1n9)	ND	0.06 ± 0.008	-
Nervonic acid (C24:1n9)	ND	0.06 ± 0.001	-
Monounsaturated fatty acid	0.56 ± 0.060	3.05 ± 0.980	<0.05
Trans-Linolelaidic acid (C18:2n6t)	ND	0.03 ± 0.003	-
Cis-9-12-Linoleic acid (C18:2n6)	ND	0.63 ± 0.002	-
gamma-Linolenic acid (C18:3n6)	ND	0.10 ± 0.004	-
alpha-Linoleic acid (C18:3n3)	ND	0.09 ± 0.001	-
Cis-11,14-Eicosadienoic acid (C20:2)	1.57 ± 0.002	0.04 ± 0.006	<0.001
Cis-8,11,14-Eicosatrienoic acid (C20:3n6)	ND	0.09 ± 0.007	-
Arachidonic acid (C20:4n6)	ND	0.05 ± 0.009	-
Cis-5,8,11,14,17-Eicosapentaenoic acid (C20:5n3)	ND	0.19 ± 0.004	-
4,7,10,13,16,19-Docosahexaenoic acid (C22:6n3)	ND	0.37 ± 0.005	-
Polyunsaturated acid	1.57 ± 0.024	1.77 ± 0.027	-
Unsaturated fat	2.13 ± 0.350	4.82 ± 0.710	<0.01
Trans fat	ND	0.13 ± 0.006	-
Omega 3 (mg/100 g)	ND	816.50 ± 65.300	-
Omega 6 (mg/100 g)	ND	872.20 ± 89.400	-
Omega 9 (mg/100 g)	563.43 ± 75.900	1990.77 ± 120.700	<0.001

Notes: ND = not detected; Values below the limits of detection (LOD) are reported as ‘ND’ and were treated as non-quantifiable in summations and reporting. LOD of 0.01 g/100 g for individual fatty acids. *p*-values have been performed by independent *t*-test (Welch’s correction).

**Table 5:** Amino acid profiles of the 4 experimental diets.

Amino Acid Compositions	Experimental Diets (mg/100 g)				*p*-Values
	T1	T2	T3	T4	
Essential amino acids					
Arginine	1244.64 ± 127.2 ^a^	1210.91 ± 134.3 ^ab^	1022.76 ± 142.4 ^ab^	870.82 ± 133.5 ^b^	0.028
Histidine	486.97 ± 62.3 ^a^	479.29 ± 53.2 ^ab^	408.75 ± 39.3 ^b^	369.08 ± 46.8 ^b^	0.031
Isoleucine	720.22 ± 52.3 ^a^	705.75 ± 54.6 ^a^	592.98 ± 47.9 ^b^	542.39 ± 65.2 ^b^	0.011
Leucine	1477.98 ± 124.8 ^a^	1453.26 ± 133.6 ^a^	1264.93 ± 142.8 ^b^	1189.39 ± 162.3 ^b^	0.046
Lysine	1015.43 ± 96.5 ^a^	994.01 ± 87.5 ^a^	803.44 ± 74.8 ^b^	642.13 ± 64.6 ^c^	<0.001
Phenylalanine	821.46 ± 88.7 ^a^	819.56 ± 59.7 ^a^	707.67 ± 87.9 ^b^	654.81 ± 55.7 ^b^	0.017
Threonine	855.21 ± 97.6 ^a^	831.71 ± 87.6 ^a^	730.71 ± 78.9 ^a^	660.23 ± 84.7 ^a^	0.055
Tryptophan	155.12 ± 18.1	167.78 ± 15.5	<150.00	<150.00	-
Valine	1002.19 ± 79.8 ^a^	977.5 ± 88.3 ^a^	844.48 ± 78.4 ^b^	781.13 ± 84.6 ^b^	0.011
Methionine	341.07 ± 24.5	323.45 ± 56.0	233.60 ± 51.2	<200	-
ΣEAA	8120.29 ± 272.69	7963.22 ± 269.41	<6609.32 ± 269.86	<5709.98 ± 268.72	-
Non-essential amino acids					
Alanine	1425.43 ± 156.3 ^a^	1338.62 ± 123.6 ^ab^	1153.64 ± 145.3 ^ab^	974.85 ± 98.34 ^b^	0.033
Aspartic acid	2215.07 ± 42.3 ^a^	2155.59 ± 32.8 ^a^	1910.62 ± 47.6 ^b^	1696.97 ± 42.9 ^c^	0.004
Glutamic acid	3270.40 ± 165.3 ^a^	3198.31 ± 132.1 ^a^	2764.79 ± 98.6 ^b^	2511.87 ± 89.7 ^c^	0.003
Glycine	1899.83 ± 85.6 ^a^	1733.73 ± 95.4 ^b^	1471.47 ± 85.6 ^c^	1168.06 ± 74.6 ^d^	<0.001
Hydroxyproline	544.82 ± 65.3	<500.00	<500.00	ND	-
Proline	1234.16 ± 59.7 ^a^	1192.50 ± 47.8 ^a^	1057.05 ± 75.9 ^b^	866.19 ± 57.9 ^c^	<0.001
Serine	978.88 ± 51.3 ^a^	962.30 ± 64.5 ^a^	843.86 ± 67.3 ^b^	782.37 ± 57.9 ^b^	0.017
Tyrosine	561.53 ± 65.9 ^a^	537.28 ± 89.4 ^a^	478.36 ± 75.5 ^a^	450.86 ± 64.7 ^a^	0.122
Cystine	200.03 ± 35.4	214.21 ± 39.7	<200	<200	-
ΣNEAA	12,330.15 ± 160.13	<11,332.54 ± 140.10	<9679.79 ± 845.90	<8451.17 ± 109.56	-

Notes: ND = not detected; LOD = limit of detection. Values below the Limits of detection (LOD) are reported as ‘ND’ and were treated as non-quantifiable in summations and reporting. LOD of hydroxyproline was 200.00 mg/100 g. Different letters in the same row indicate significant differences (*p* < 0.05).

**Table 6:** Fatty acid profiles of the 4 experimental diets.

Fatty Acid Compositions	Experimental Diets (g/100 g)				*p*-Values
	T1	T2	T3	T4	
Lauric acid (C12:0)	0.01 ± 0.002	0.01 ± 0.001	0.01 ± 0.002	ND	-
Myristic acid (C14:0)	0.13 ± 0.002 ^a^	0.10 ± 0.003 ^b^	0.09 ± 0.001 ^c^	0.05 ± 0.002 ^d^	<0.001
Pentadecanoic acid (C15:0)	0.04 ± 0.001 ^a^	0.03 ± 0.003 ^b^	0.03 ± 0.004 ^b^	0.01 ± 0.001 ^c^	<0.001
Palmitic acid (C16:0)	1.62 ± 0.042 ^a^	1.57 ± 0.034 ^ab^	1.48 ± 0.024 ^b^	1.22 ± 0.060 ^c^	<0.001
Heptadecanoic acid (C17:0)	0.06 ± 0.002 ^a^	0.04 ± 0.003 ^b^	0.04 ± 0.004 ^b^	0.03 ± 0.001 ^c^	<0.001
Stearic acid (C18:0)	0.54 ± 0.020 ^a^	0.49 ± 0.030 ^ab^	0.47 ± 0.030 ^ab^	0.43 ± 0.050 ^b^	0.026
Saturate fat	2.43 ± 0.047 ^a^	2.26 ± 0.046 ^b^	2.15 ± 0.039 ^b^	1.76 ± 0.070 ^c^	<0.001
Palmitoleic acid (C16:1n7)	0.15 ± 0.021 ^a^	0.10 ± 0.022 ^ab^	0.10 ± 0.015 ^ab^	0.05 ± 0.024 ^b^	0.003
Cis-9-Oleic acid (C18:1n9c)	1.58 ± 0.120 ^a^	1.49 ± 0.140 ^a^	1.41 ± 0.130 ^a^	1.23 ± 0.210 ^a^	0.107
Trans-9-Elaidic acid (C18:1n9t)	0.01 ± 0.001	ND	ND	ND	
Cis-11-Eicosenoic acid (C20:1n11)	0.08 ± 0.002 ^a^	0.06 ± 0.001 ^b^	0.06 ± 0.002 ^b^	0.03 ± 0.001 ^c^	<0.001
Erucic acid (C12:1n9)	0.01 ± 0.001	ND	ND	ND	
Nervonic acid (C24:1n9)	0.05 ± 0.002 ^a^	0.05 ± 0.003 ^a^	0.04 ± 0.002 ^b^	0.04 ± 0.004 ^b^	0.003
Monounsaturated fatty acid	1.87 ± 0.122 ^a^	1.71 ± 0.142 ^ab^	1.63 ± 0.131 ^ab^	1.36 ± 0.211 ^b^	0.022
Cis-9-12-Linoleic acid (C18:2n6)	0.83 ± 0.120 ^a^	0.71 ± 0.210 ^a^	0.65 ± 0.230 ^a^	0.56 ± 0.110 ^a^	0.356
gamma-Linolenic acid (C18:3n6)	0.05 ± 0.002 ^a^	0.04 ± 0.004 ^b^	0.04 ± 0.001 ^b^	0.04 ± 0.003 ^b^	0.005
alpha-Linoleic acid (C18:3n3)	0.03 ± 0.001 ^a^	0.03 ± 0.004 ^a^	0.03 ± 0.003 ^a^	0.02 ± 0.005 ^b^	0.020
Cis-11,14-Eicosatrienoic acid (C20:2)	0.01 ± 0.002	ND	ND	ND	-
Cis-8,11,14-Eicosatrienoic acid (C20:3n6)	0.04 ± 0.003 ^a^	0.03 ± 0.001 ^b^	0.03 ± 0.002 ^b^	0.02 ± 0.005^c^	<0.001
Cis-5,8,11,14,17-Eicosapentaenoic acid (C20:5n3)	0.05 ± 0.003	0.03 ± 0.002	0.02 ± 0.001	ND	-
4,7,10,13,16,19-Docosahexaenoic acid (C22:6n3)	0.09 ± 0.002 ^a^	0.05 ± 0.003 ^b^	0.03 ± 0.002 ^c^	0.01 ± 0.005 ^d^	<0.001
Polyunsaturated acid	1.30 ± 0.120 ^a^	1.10 ± 0.210 ^ab^	1.00 ± 0.230 ^ab^	0.65 ± 0.110 ^b^	0.011
Unsaturated fat	3.030 ^a^	2.830 ^b^	2.760 ^b^	2.030 ^c^	<0.001
Tran fat	0.020	0.010	0.020	ND	-
Omega 3 (mg/100 g)	217.83 ± 21.2 ^a^	131.55 ± 31.0 ^b^	88.53 ± 24.5 ^bc^	42.95 ± 20.6 ^c^	<0.001
Omega 6 (mg/100 g)	996.67 ± 62.3 ^a^	863.25 ± 76.8 ^ab^	733.04 ± 76.3 ^bc^	621.66 ± 67.3 ^c^	0.001
Omega 9 (mg/100 g)	1725.27 ± 135.1 ^a^	1618.96 ± 198.7 ^ab^	1556.36 ± 164.2 ^ab^	1275.16 ± 143.6 ^b^	0.048

Notes: ND = not detected; LOD = limit of detection. Values below the Limits of detection (LOD) are reported as ‘ND’ and were treated as non-quantifiable in summations and reporting. LOD of 0.01 g/100 g for individual fatty acids. Different letters in the same row indicate significant differences (*p* < 0.05).

**Table 7:** Amino acid composition in fillet obtained each experimental group.

Amino Acid Compositions	Amino Acid Content in Fish Muscles Fed with Each Diet (mg/100 g)				*p*-Values
	T1	T2	T3	T4	
Essential amino acids (EAA)					
Arginine	1066.96 ± 96.3	1063.31 ± 84.5	1050.98 ± 89.6	975.57 ± 56.2	0.525
Histidine	456.03 ± 75.3	444.81 ± 68.9	431.77 ± 78.4	411.62 ± 56.9	0.816
Isoleucine	759.04 ± 89.7	759.91 ± 87.1	744.95 ± 76.9	694.83 ± 88.3	0.607
Leucine	1445.123 ± 59.8	1445.14 ± 118.1	1437.53 ± 133.5	1333.54 ± 128.2	0.477
Lysine	1654.78 ± 124.6	1647.42 ± 143.2	1678.08 ± 104.3	1517.23 ± 146.7	0.480
Phenylalanine	751.00 ± 68.7	755.32 ± 87.3	740.72 ± 98.2	743.90 ± 67.2	0.919
Threonine	809.05 ± 87.3	807.60 ± 56.8	804.70 ± 65.8	749.97 ± 75.9	0.586
Tryptophan	<150	<150	<150	<150	-
Valine	852.63 ± 86.5	859.16 ± 89.4	843.20 ± 76.9	788.53 ± 68.7	0.638
Methionine	407.77 ± 56.3	394.71 ± 59.2	416.41 ± 45.8	319.63 ± 89.7	0.297
ΣEAA	8202.89 ± 255.11 ^a^	8177.38 ± 276.17 ^a^	8148.34 ± 266.00 ^a^	7534.82 ± 274.24 ^b^	0.031
Non-essential amino acids (NEAA)					
Alanine	1094.85 ± 132.0	1113.16 ± 121.5	1112.58 ± 143.2	1018.96 ± 98.7	0.757
Aspartic acid	2067.86 ± 168.3	2042.29 ± 154.8	2086.64 ± 157.6	1898.07 ± 157.9	0.485
Glutamic acid	2929.05 ± 124.5	2920.40 ± 104.7	2910.96 ± 139.8	2708.32 ± 163.7	0.209
Glycine	1062.37 ± 98.6	1087.36 ± 87.2	1012.76 ± 69.7	972.69 ± 87.1	0.388
Hydroxyproline	ND	ND	ND	ND	-
Proline	577.95 ± 65.9	593.88 ± 74.2	561.22 ± 81.8	541.07 ± 77.5	0.791
Serine	732.81 ± 65.2	731.12 ± 98.2	746.72 ± 75.9	675.29 ± 58.7	0.667
Tyrosine	583.03 ± 88.7	668.98 ± 79.5	681.12 ± 69.8	626.01 ± 98.5	0.423
Cystine	ND	ND	ND	ND	-
ΣNEAA	9047.92 ± 295.68	9157.19 ± 280.64	9112.00 ± 295.07	8440.41 ± 297.01	0.054

Notes: ND = not detected; LOD = limit of detection. Values below the Limits of detection (LOD) are reported as ‘ND’ and were treated as non-quantifiable in summations and reporting. LOD of hydroxyproline was 200.00 mg/100 g. LOD were: cystine, 100.00 mg/100 g and hydroxyproline 200.00 mg/100 g. Each value represents the mean ± SD of three replicate cages per treatment (*n* = 3). Different letters in the same row indicate significant differences (*p* < 0.05).

**Table 8:** Descriptive alpha-diversity indices of pooled intestinal microbiota profiles from red tilapia receiving different dietary formulations containing graded levels of *Azolla microphylla* meal.

Sample ID	Feature	ACE	Chao1	Simpson	Shannon	PD_Whole_Tree	Coverage
Neg-Ctr	107	107.6984	107.0370	0.6805	2.4077	6.0061	1.0
T1B	47	47.0	47.0	0.3024	0.874	2.2975	1.0
T1F	87	87.6061	87.05	0.6264	2.584	4.3103	1.0
T2B	972	972.4476	972.0	0.9911	8.138	45.7914	1.0
T2F	40	40.7714	40.0833	0.6631	2.3995	2.988	1.0
T3B	57	58.9297	57.5455	0.7047	2.3554	3.4055	0.9999
T3F	161	161.6569	161.0263	0.2704	1.1049	6.4256	1.0
T4B	79	79.3779	79.0	0.5895	1.5555	3.9231	1.0
T4F	615	616.1127	615.0606	0.9516	5.8823	14.4527	0.9999

Note: For T1–T4, each entry represents one pooled intestinal sample containing material from five fish. Values describe individual pooled samples and are not treatment means or estimates of between-fish variability. No inferential statistical comparisons were performed. The negative control (Neg-Ctr) represents a subgroup of T1 fish injected with sterile physiological saline (0.85% NaCl) instead of *A. hydrophila* and is included as a descriptive reference only. It was not part of the dietary treatment comparisons.

## Data Availability

The data presented in this study are available on request from the corresponding author (please specify the reason for restriction, e.g., the data are not publicly available due to privacy or ethical restrictions).

## Abbreviations

The following abbreviations are used in this manuscript:

ADG	Average daily gain
ALP	Alkaline phosphatase
ALT	Alanine transaminase
AMM	*Azolla microphylla* meal
ANOVA	Analysis of variance
AOAC	Association of Official Analytical Chemists
ASVs	Amplicon sequence variants
AST	Serum aspartate aminotransferase
CFM	Commercial fish meal
CFU	Colony forming unit
CRD	Completely randomized design
DHA	Docosahexaenoic acid
DO	Dissolved oxygen
EAA	Essential amino acid
EPA	Eicosapentaenoic acid
FCR	Feed conversion ratio
GC	Gas chromatography
KEGG	Kyoto Encyclopedia of Genes and Genomes
LOD	Limit of detection
MS-222	Tricaine methane sulfonate
MUFA	Monounsaturated fatty acid
NEAA	Non-essential amino acid
Neg-Ctr	Negative control
PBS	Phosphate-buffered saline
PCR	Polymerase chain reaction
PICRUSt2	Phylogenetic Investigation of Communities by Reconstruction of Unobserved States 2
PUFA	Polyunsaturated fatty acid
RDP	Ribosomal Database Project
SBM	Soybean meal
SFA	Saturated fatty acid
SGR	Specific growth rate
SR	Survival rate
TSB	Tryptic Soy Broth
UPGMA	Unweighted Pair Group Method with Arithmetic Mean
WG	Weight gain

## References

[1] Mejía G.C., Mejía J.C., Navarrete K.A.S., Martínez A.M.M., Castellón A.E.C. (2023). Comparison of growth (length and weight) gain, TCA, TIC, KM, and feed efficiency of Pargo tilapia, UNAM cultured in biofloc at 10 g L−1 salinity at different densities. Int. J. Fish. Aquat. Stud..

[2] Cortés-Sánchez A.D.J. (2021). About Clostridium botulinum, fish and tilapia. Mod. Appl. Sci..

[3] Daudpota A.M., Kalhoro I.B., Shah S.A., Kalhoro H., Abbas G. (2014). Effect of stocking densities on growth, production and survival rate of red tilapia in hapa at fish hatchery Chilya Thatta, Sindh, Pakistan. J. Fish..

[4] Mohamad S.N., Noordin W.N.M., Ismail N.F., Hamzah A. (2021). Red hybrid tilapia (*Oreochromis* spp.) broodstock development programme in Malaysia: Status, challenges and prospects for future development. Asian Fish. Sci..

[5] Debnath S.C., McMurtrie J., Temperton B., Delamare-Deboutteville J., Mohan C.V., Tyler C.R. (2023). Tilapia aquaculture, emerging diseases, and the roles of the skin microbiomes in health and disease. Aquac. Int..

[6] Wu L., Yang J. (2012). Identifications of captive and wild tilapia species existing in Hawaii by mitochondrial DNA control region sequence. PLoS ONE.

[7] Magouz F.I., Dawood M.A.O., Salem M.F.I., Mohamed A.A.I. (2020). The effects of fish feed supplemented with Azolla meal on the growth performance, digestive enzyme activity, and health condition of genetically improved farmed tilapia (*Oreochromis niloticus*). Ann. Anim. Sci..

[8] Namiq K., Mahmood S.S., Abdulrahman N.M., Hassan B.R., Sadraddin A.A., Rashed R.M. (2021). Effect of feeding common carp (*Cyprinus carpio* (Linnaeus, 1758)) with water fern (*Azolla filiculoides* (Lam.)) on its fillet quality. Ribogospod. Nauka Ukr..

[9] Chepkirui M., Orina P.S., Opiyo M., Muendo P., Mbogo K., Omondi R. (2022). Growth performance of Nile tilapia (*Oreochromis niloticus*) fingerlings fed with water spinach (*Ipomoea aquatica*) diets. Ann. Mar. Sci..

[10] Moyo N.A.G., Rapatsa-Malatji M.M. (2023). A review and meta-analysis of selected plant protein sources as a replacement of fishmeal in the diet of tilapias. Ann. Anim. Sci..

[11] Gaidhane S., Ahilan B., Chidambaram P., Jayakumar N. (2023). Azolla meal as an alternative protein source in the diet of *Etroplus suratensis*. Res. Sq..

[12] Nephale L.E., Moyo N.A., Rapatsa-Malatji M.M. (2024). Partial replacement of fish meal with soldier termite in juvenile Mozambique tilapia: Effects on growth performance, blood serum chemistry and histomorphology. J. Anim. Feed Sci..

[13] Kiziloğlu Ü., Yıldırım Ö., Çantaş İ.B. (2023). Use of coontail as a natural phytoremediation feed additive for common carp. Oceanol. Hydrobiol. Stud..

[14] Caruso D., Lusiastuti A.M., Pouil S., Samsudin R., Arifin O.Z., Slembrouck J. (2023). Can Azolla filiculoides be a complementary feed resource for ecological intensification in small-scale fish farming? Biological effects on giant gourami (*Osphronemus goramy*). Aquat. Living Resour..

[15] Mansour A.T., Alsaqufi A.S., Alkhamis Y.A., Al-Gazar F.F., Zaki M.A., Nour A.A.M., Ramadan K.M.A. (2021). The evaluation of Arthrospira platensis bioactivity and its dietary supplementation to a vegetarian diet of Nile tilapia on growth performance, feed utilization, body composition and hemato-biochemical parameters. Ann. Anim. Sci..

[16] AOAC (2006). Official Methods of Analysis.

[17] Official Journal of the European Communities (1998). Determination of amino acids. Off. J. Eur. Communities.

[18] AOAC (2019). Official Methods of Analysis.

[19] Bagenal T. (1978). Methods for Assessment of Fish Production in Fresh Waters.

[20] Illumina (2013). 16S Metagenomic Sequencing Library Preparation.

[21] Bolger A.M., Lohse M., Usadel B. (2014). Trimmomatic: A flexible trimmer for Illumina sequence data. Bioinformatics.

[22] Martin M. (2011). Cutadapt removes adapter sequences from high-throughput sequencing reads. EMBnet J..

[23] Callahan B.J., McMurdie P.J., Rosen M.J., Han A.W., Johnson A.J.A., Holmes S.P. (2016). DADA2: High-resolution sample inference from Illumina amplicon data. Nat. Methods.

[24] RStudio Team (2020). RStudio: Integrated Development for R.

[25] Wang Q., Garrity G.M., Tiedje J.M., Cole J.R. (2007). Naive Bayesian classifier for rapid assignment of rRNA sequences into the new bacterial taxonomy. Appl. Environ. Microbiol..

[26] Estaki M., Jiang L., Bokulich N.A., McDonald D., González A., Kosciolek T., Martino C., Zhu Q., Birmingham A., Vázquez-Baeza Y. (2020). QIIME 2 enables comprehensive end-to-end analysis of diverse microbiome data and comparative studies with publicly available data. Curr. Protoc. Bioinform..

[27] Douglas G.M., Maffei V.J., Zaneveld J.R., Yurgel S.N., Brown J.R., Taylor C.M., Huttenhower C., Langille M.G.I. (2020). PICRUSt2 for prediction of metagenome functions. Nat. Biotechnol..

[28] Kanehisa M., Goto S. (2000). KEGG: Kyoto encyclopedia of genes and genomes. Nucleic Acids Res..

[29] Nasir N.A.N.M., Kamaruddin S.A., Zakarya I.A., Aminul Islam A.K.M. (2022). Sustainable alternative animal feeds: Recent advances and future perspective of using Azolla as animal feed in livestock, poultry and fish nutrition. Sustain. Chem. Pharm..

[30] Camargo T.R., Bueno G.W., Machado L.P., Brande M.R., Heitzman B.S., Trombeta T.D. (2023). Bioprocess for mass production and feed utilization of Azolla pinnata in aquaculture ponds: A perspective of bioeconomy and eco-friendly technology for small farms. Rev. Bras. Eng. Biossist..

[31] Chandrababu K.A., Parvathy U., Meenu B., Binsi P.K. (2024). Unveiling the nutritional treasure trove: A comparative analysis of two Azolla species for potential food applications. Food Humanit..

[32] Naseem S., Bhat S.U., Gani A., Bhat F.A. (2023). Towards understanding the prospects of select macrophytes as potential fish feed. Res. Sq..

[33] Ndeda V. (2022). Exploring genus Amaranthus as a promising fishmeal alternative in aquaculture: A mini review. Int. J. Fish. Aquat. Stud..

[34] Seephueak W., Sangkaew B., Cherdthong A., Khonkhaeng B., Supapong C. (2025). Development of animal feed from *Azolla microphylla* to increase production efficiency of goat farms in southern Thailand. BMC Vet. Res..

[35] Egerton S., Wan A., Murphy K., Collins F., Ahern G., Sugrue I., Busca K., Egan F., Muller N., Whooley J. (2020). Replacing fishmeal with plant protein in Atlantic salmon (*Salmo salar*) diets by supplementation with fish protein hydrolysate. Sci. Rep..

[36] Li X., Wang S., Zhang M., Jiang H., Qian Y., Wang R., Li M. (2023). Comprehensive analysis of metabolomics on flesh quality of yellow catfish (*Pelteobagrus fulvidraco*) fed plant-based protein diet. Front. Nutr..

[37] Stoneham T.R., Kuhn D.D., Taylor D.P., Neilson A.P., Smith S.A., Gatlin D.M., Chu H.S.S., O’Keefe S.F. (2018). Production of omega-3 enriched tilapia through the dietary use of algae meal or fish oil: Improved nutrient value of fillet and offal. PLoS ONE.

[38] Kousoulaki K., Sveen L., Norén F., Espmark Å. (2022). Atlantic salmon (*Salmo salar*) performance fed low trophic ingredients in a fish meal and fish oil free diet. Front. Physiol..

[39] Mastoraki M., Mollá Ferrándiz P.M., Vardali S.C., Kontodimas D.C., Kotzamanis Y.P., Gasco L., Chatzifotis S., Antonopoulou E. (2020). A comparative study on the effect of fish meal substitution with three different insect meals on growth, body composition and metabolism of European sea bass *(Dicentrarchus labrax* L.). Aquaculture.

[40] Yohana M.A., Ray G.W., Yang Q., Tan B., Chi S., Asase A., Shiyu K., Derrick A. (2023). A review on the use of Azolla meal as a feed ingredient in aquatic animals’ diets. Aquac. Res..

[41] Ahmed I., Khan Y.M., Lateef A., Majeed A., Shah M.A. (2022). Partial replacement of fishmeal by Azolla cristata in diets for fingerling common carp, *Cyprinus carpio* var. communis. Res. Sq..

[42] Minich J.J., Michael T.P. (2024). A review of using duckweed (Lemnaceae) in fish feeds. Rev. Aquac..

[43] Upaskar S.S., Tibile R.M., Pawase A.S., Ghode G.S., Bhatkar V.R., Tijare S.K., Chikate S.D., Charthad Y.V. (2024). Growth and survival of Pearlspot, *Etroplus suratensis* (Bloch, 1790), fry using practical diets incorporated with different levels of mosquito fern. Indian J. Anim. Sci..

[44] Mosha S.S. (2018). A review on significance of Azolla meal as a protein plant source in finfish culture. J. Aquac. Res. Dev..

[45] Ahirwal S.K., Sarma K., Dey A., Kumar T., Bharti V., Singh J. (2024). Investigations on growth performance of grass carp *Ctenopharyngodon idella* (Cuvier and Valenciennes, 1844) under forage crops and aquatic macrophyte feeding regimes. Indian J. Fish..

[46] Fiordelmondo E., Ceschin S., Magi G.E., Mariotti F., Iaffaldano N., Galosi L., Roncarati A. (2022). Effects of partial substitution of conventional protein sources with duckweed (*Lemna minor*) meal in the feeding of rainbow trout (Oncorhynchus mykiss) on growth performances and the product quality. Plants.

[47] Ahmed I., Khan Y.M., Lateef A., Jan K., Majeed A., Shah M.A. (2023). Effect of fish meal replacement by Azolla meal on growth performance, hemato-biochemical and serum parameters in the diet of scale carp, *Cyprinus carpio* var. communis. J. World Aquac. Soc..

[48] Belagoune A.N.E., Beghoul S., Djemai S., Hafid K., Amireche D., Mansour A. (2024). The impact of Azolla (*Azolla pinnata* R. Br.) dietary supplementation on broiler chicken production performance and serum biochemical profile. Rev. Fac. Agron..

[49] El-Fadel A., Hassanein H.A.M., El-Sanafawy H.A. (2020). Effect of partial replacement of protein sun flower meal by Azolla meal as source of protein on productive performance of growing lambs. J. Anim. Poult. Prod..

[50] Shaima S.M., Bakhan R.H., Karzan F.N., Rabar M.R., Avan A.S., Nasreen M.A. (2020). Impact of natural Azolla filiculoides powders on some physiological, nutritional and biological parameters of common carp (*Cyprinus carpio* L.). Appl. Ecol. Environ. Res..

[51] Wang B., Zhang X., Liu Y., Gao M., Wang M., Wang Y., Wang X., Guo Y. (2024). Assessment of the dietary amino acid profiles and the relative biomarkers for amino acid balance in low-protein diets for broiler chickens. J. Anim. Sci. Biotechnol..

[52] Brezas A., Hardy R.W. (2020). Improved performance of a rainbow trout selected strain is associated with protein digestion rates and synchronization of amino acid absorption. Sci. Rep..

[53] de Francesco M., Parisi G., Médale F., Lupi P., Kaushik S.J., Poli B.M. (2004). Effect of long-term feeding with a plant protein mixture based diet on growth and body/fillet quality traits of large rainbow trout (Oncorhynchus mykiss). Aquaculture.

[54] Qi X., Zhang Y., Zhang Y., Luo F., Song K., Wang G., Ling F. (2023). Vitamin B12 produced by *Cetobacterium somerae* improves host resistance against pathogen infection through strengthening interactions within the gut microbiota. Microbiome.

[55] Ofek T., Lalzar M., Laviad-Shitrit S., Izhaki I., Halpern M. (2021). Comparative study of intestinal microbiota composition of six edible fish species. Front. Microbiol..

[56] Pérez-Pascual D., Pérez-Cobas A.E., Rigaudeau D., Rochat T., Bernardet J.F., Skiba-Cassy S., Marchand Y., Duchaud E., Ghigo J.M. (2021). Sustainable plant-based diets promote rainbow trout gut microbiota richness and do not alter resistance to bacterial infection. Anim. Microbiome.

[57] Korsa G., Alemu D., Ayele A. (2024). Azolla Plant Production and Their Potential Applications. Int. J. Agron..

